# Modeling risk dependence and portfolio VaR forecast through vine copula for cryptocurrencies

**DOI:** 10.1371/journal.pone.0242102

**Published:** 2020-12-23

**Authors:** Khreshna Syuhada, Arief Hakim

**Affiliations:** Statistics Research Division, Institut Teknologi Bandung, Bandung, Indonesia; Universidad de Salamanca, SPAIN

## Abstract

Risk in finance may come from (negative) asset returns whilst payment loss is a typical risk in insurance. It is often that we encounter several risks, in practice, instead of single risk. In this paper, we construct a dependence modeling for financial risks and form a portfolio risk of cryptocurrencies. The marginal risk model is assumed to follow a heteroscedastic process of GARCH(1,1) model. The dependence structure is presented through vine copula. We carry out numerical analysis of cryptocurrencies returns and compute Value-at-Risk (VaR) forecast along with its accuracy assessed through different backtesting methods. It is found that the VaR forecast of returns, by considering vine copula-based dependence among different returns, has higher forecast accuracy than that of returns under prefect dependence assumption as benchmark. In addition, through vine copula, the aggregate VaR forecast has not only lower value but also higher accuracy than the simple sum of individual VaR forecasts. This shows that vine copula-based forecasting procedure not only performs better but also provides a well-diversified portfolio.

## Introduction

In finance and insurance, one of the major and challenging issues is managing quantitative risk, specifically forecasting future risk. Risk forecast is not only important for reserving capital but also for anticipating the worse risk. Risk in finance may come from (negative) asset returns whilst, in insurance, a typical risk is a payment loss. It is often that we encounter several (dependent) risks, in practice, instead of a single risk. Dependent random risks or losses occur in many applications and have challenging statistical features, see e.g. Embrechts et al. [1, 2], Gräler et al. [3], McNeil et al. [4], Naifar [5], Patton [6], Trabelsi [7], Usman et al. [8] and Zhang et al. [9].

There are several interesting topics that relate to dependent risks. The first one is construction of dependent risks either as an aggregate risk model or a multivariate risk model, see e.g. Kim and Kim [10]. The second topic lies on the fact that dependent risks have a technical problem with regard to finding an exact form of distribution function (cdf) or probability function (pdf). Both topics above eventually bring us to learn and employ a more sophisticated method in dependent risks namely copula. Copula is a system that does not only accommodate either non normal or unidentical marginal distributions into a uniform distribution but also simplify number of parameters of a joint distribution. Meanwhile, a more step method than a copula is vine copula. It is also a system that constructs pair-copula with high flexibility when decomposing conditional distribution, e.g. Aas et al. [11] and Trucíos et al. [12]. Furthermore, vine copula provides us informative and complete dependence structure. It is well known that understanding dependence is an important step to make strategy on diversification.

Modeling dependence through vine copula approach is basically aimed to broaden flexibility of dependence structure among random risks, compared to classical dependence measures of Pearson’s *ρ* and Kendall’s *τ* or even copula. The recent use of vine copula modeling may be found in (energy) economic and finance applications. For instance, Mejdoub and Ghorbel [13] investigated conditional dependence between oil price and renewable energy stock prices and considered threshold Generalized Autoregressive Conditional Heteroscedastic (GARCH) model, see also Trabelsi [7] for tail risk dependence between oil and stocks of oil-exporting countries. Kumar et al. [14] examined conditional dependence among not only energy commodities but also agricultural and precious metals commodities. Furthermore, Çekin et al. [15] studied dependence structure among economic policy uncertainty (EPU) of Latin American countries. Meanwhile, Hernandez et al. [16] compared risk of portfolio: Gulf Cooperation Council (GCC) Islamic and conventional bank indices. They studied tail asymmetric dependence among Islamic banks’ relationship. In addition, Usman et al. [8] explored dependence modeling between Islamic and conventional stocks through copula whilst Naifar [5] employed Archimedean copulas to model tail dependence structure between Islamic bonds and stock market.

This paper considers risk in finance defined as (negative) asset returns. In particular, we construct a dependence modeling for financial risks and form a portfolio risk. Whilst marginal risk is assumed to follow a Generalized Autoregressive Conditional Heteroscedastic (GARCH) model of order one, forecasting future risk is carried out by risk measure of Value-at-Risk (VaR). VaR, along with Conditional VaR, has be applied to both single risk and dependent risks to make use in practice, see e.g. Nieto and Ruiz [17] for latest review on VaR and its backtesting. Basically, VaR is a maximum tolerated risk or loss for either single or aggregate risk at a given level of confidence. VaR forecast is crucial for assessing the performance of financial institution. Embrechts et al. [1, 2] emphasized that VaR is the industry and regulatory standard for risk capital calculation in both banking and insurance.

We pay particular attention to portfolio risk of cryptocurrencies returns; particularly, Bitcoin (BTC), Ethereum (ETH) and Litecoin (LTC) are considered. Cryptocurrency has been one of the major interests among financial practitioners, investors, academia even policy makers. Bitcoin, since introduced by Nakamoto [18], has shown a dramatic increasing value during a year period of 2017 and 2018 for about one and a half times ([19]). It has been a prominent digital asset ever since. However, there is still debate whether cryptocurrencies are defined as currency, commodity or investment asset. Jiménez et al. [20] argued that Bitcoin is a digital asset or investment as known before (bonds and equities). Their interests are on volatility clustering and leptokurtosis which are typical in asset returns. However, we may observe an extreme event on BTC that leads to greater market instability. There are some financial implications on cryptocurrencies due to uncontrolled monitoring by monetary regulator, see. e.g. Corbet et al. [21] who recorded pricing bubbles in BTC and ETH. They also claimed that cryptocurrencies may become source of financial instability.

Boako et al. [19] and Trucíos et al. [12] are among authors recently used returns of cryptocurrencies to illustrate vine copula modeling and risk measures forecasting. It is interesting to classify several areas done by authors. The first area is cryptocurrencies testing in order to have a clear position: as a currency, commodity or asset; see e.g. White et al. [22] and Kwon [23]. The related area to the first is pricing formation of cryptocurrencies and other possible measurement such as ratio of gold to platinum (GP) prices forecast Bitcoin return, e.g. Huynh et al. [24]. The second area lies on cryptocurrencies empirical facts like other returns along with return-volatility correlation, volatility clustering or asymmetric volatility, see e.g. Bouri et al. [25], Klein et al. [26], Boako et al. [19] and Wajdi et al. [27]. Such relationship may be measured by what so called “volatility surprise” or unexpected volatility. It is the difference between squared innovation and conditional volatility, see Bouri et al. [28]. Furthermore, interdependence that accounts time and frequency and market interconnection among cryptocurrencies may be explored through wavelet-based approaches CWT, continuous wavelet transformation, and XWT, cross wavelet transform) as carried out by e.g. Qureshi et al. [29]. Interdependence may also be observed between cryptocurrency and energy or agricultural commodities. Ji et al. [30], for instance, examined information spillovers via entropy-based method among both cryptocurrencies and commodities. They found that, first, it changes over time. Secondly, unlike the spillover of cryptocurrencies, energy commodities spillover contribution to the system is dependent on their price dynamics. The third area is concerned about economic or financial implication of cryptocurrencies, see e.g. Bouri et al. [31] who studied global financial stress, whilst Trucíos et al. [12] argued that it is a shelter against economic and financial turmoil.

In this paper, we show the portfolio or aggregate VaR forecast by considering vine copula-based dependence among individual returns. Different from the work of Boako et al. [19] and Trucíos et al. [12] who found VaR forecast by using classical historical simulation (HS) method, our forecast calculation considers “estimative” VaR forecast as in Kabaila and Syuhada [32, 33] and Syuhada [34]. The aggregate VaR forecast is compared to the simple sum of individual VaR forecasts that actually considers perfect dependence assumption. To evaluate their performance, we assess their accuracy by adopting several backtesting methods as recently used by Syuhada [34] and Jiménez et al. [20]. In addition, comparing the aggregate VaR and the simple sum of individual VaRs also leads us to investigate and measure benefits of the portfolio diversification.

## Material and methods

### Data

Cryptocurrencies data, Bitcoin (BTC), Ethereum (ETH) and Litecoin (LTC), are obtained from Coin Market Cap (coinmarketcap.com) for period 1 January 2017 till 31 December 2018 (730 days).

### Returns and marginal risk model

The marginal risk is (negative) returns of three cryptocurrencies defined as below

$$\begin{array}{c} X_{t}=-\ln(\frac{P_{t}^{B}}{P_{t-1}^{B}}),\;Y_{t}=-\ln(\frac{P_{t}^{E}}{P_{t-1}^{E}}),\;Z_{t}=-\ln(\frac{P_{t}^{L}}{P_{t-1}^{L}}), \end{array}$$ (1)

where $P_{t}^{B}$, $P_{t}^{E}$ and $P_{t}^{L}$ denote (closing) price at time *t* for Bitcoin (BTC), Ethereum (ETH) and Litecoin (LTC), respectively.

We assume a marginal risk model of Generalized Autoregressive Conditional Heteroscedastic [35] of order one or GARCH(1,1) for each risk process. This is mainly due to dynamic volatility property found from each risk. Specifically, the GARCH(1,1) models for (negative) returns of Bitcoin {*X*_*t*_}, Ethereum {*Y*_*t*_} and Litecoin {*Z*_*t*_} are, respectively, given by

$$\begin{array}{c} \begin{array}{cc} X_{t} & =\sigma_{x;t}\;\varepsilon_{x;t},\;\sigma_{x;t}^{2}=\omega_{x}+\delta_{x}\;X_{t-1}^{2}+\beta_{x}\;\sigma_{x;t-1}^{2};\\ Y_{t} & =\sigma_{y;t}\;\varepsilon_{y;t},\;\sigma_{y;t}^{2}=\omega_{y}+\delta_{y}\;Y_{t-1}^{2}+\beta_{y}\;\sigma_{y;t-1}^{2};\\ Z_{t} & =\sigma_{z;t}\;\varepsilon_{z;t},\;\sigma_{z;t}^{2}=\omega_{z}+\delta_{z}\;Z_{t-1}^{2}+\beta_{z}\;\sigma_{z;t-1}^{2}; \end{array} \end{array}$$ (2)

where *ω*_*x*_, *ω*_*y*_, *ω*_*z*_ > 0 and *δ*_*x*_, *δ*_*y*_, *δ*_*z*_, *β*_*x*_, *β*_*y*_, *β*_*z*_ ≥ 0, for *t* ≥ 0. The restriction on persistence parameter *δ*_*x*_ + *β*_*x*_ < 1, *δ*_*y*_ + *β*_*y*_ < 1 and *δ*_*z*_ + *β*_*z*_ < 1 is needed to ensure the stationarity of all processes {*X*_*t*_}, {*Y*_*t*_} and {*Z*_*t*_}, respectively. We assume that innovation {*ε*_*x*;*t*_, *t* ≥ 0} is white noise. In addition, innovation *ε*_*x*;*t*_, *t* ≥ 0, and volatility *σ*_*x*;*t*_ as well as *ε*_*x*;*t*_, *t* ≥ 0, and information, up to time (*t* − 1), $F_{x;t-1}$, are independent. Note that such assumptions also apply to {*ε*_*y*;*t*_, *t* ≥ 0} and {*ε*_*z*;*t*_, *t* ≥ 0}.

The estimates for each innovation is calculated and the goodness-of-fit procedure for innovation distribution needs to be carried out. From *X*_*t*_ = *σ*_*x*;*t*_ *ε*_*x*;*t*_, the innovation *ε*_*x*;*t*_ is formulated as *ε*_*x*;*t*_ = *X*_*t*_/*σ*_*x*;*t*_. Thus, *ε*_*x*;*t*_ may be estimated by

$$\begin{array}{c} {\hat{\varepsilon }}_{x;t}=\frac{x_{t}}{{\hat{\sigma }}_{x;t}},\;{\hat{\sigma }}_{x;t}=\sqrt{\frac{1}{t-1}\sum_{s=1}^{t}{\left(x_{s}-\frac{1}{t}\sum_{r=1}^{t}x_{r}\right)}^{2}}. \end{array}$$ (3)

Meanwhile, we assume standard Student’s *t* distribution for such innovation. To do so, suppose that a random variable *T*_*x*_ has Student’s *t* distribution with degrees of freedom *ν*_*x*_ ∈ (0, ∞), i.e. *T*_*x*_ ∼ *t*(*ν*_*x*_). The probability function of *T*_*x*_ is

$$\begin{array}{c} f_{T_{x}}\left(t\right)=\frac{1}{\sqrt{\nu_{x}}\;B\left(\frac{\nu_{x}}{2},\frac{1}{2}\right)}{\left(1+\frac{t^{2}}{\nu_{x}}\right)}^{-\frac{\nu_{x}+1}{2}},\;t\in R, \end{array}$$

where *B*(⋅, ⋅) is beta function. Note that its mean is $E(T_{x})=0$, for *ν*_*x*_ > 1, whilst its variance, $V\left(T_{x}\right)=\frac{\nu_{x}}{\nu_{x}-2}$, is positive and finite, for *ν*_*x*_ > 2. By defining

$$\begin{array}{c} \varepsilon_{x;t}=\frac{T_{x}}{\sqrt{V(T_{x})}}=T_{x}\sqrt{\frac{\nu_{x}-2}{\nu_{x}}}, \end{array}$$

we obtain $E(\varepsilon_{x;t})=0$ and $V(\varepsilon_{x;t})=1$ and the innovation *ε*_*x*;*t*_ is said to have standard Student’s *t* distribution with probability function

$$\begin{array}{c} f_{\varepsilon_{x}}\left(\varepsilon \right)=\frac{1}{\sqrt{\nu_{x}-2}\;B\left(\frac{\nu_{x}}{2},\frac{1}{2}\right)}{\left(1+\frac{\varepsilon^{2}}{\nu_{x}-2}\right)}^{-\frac{\nu_{x}+1}{2}},\;\varepsilon \in R. \end{array}$$

Parameter function $\sqrt{\frac{\nu_{x}-2}{\nu_{x}}}$ may be viewed as scale parameter, so that

$$\begin{array}{c} \varepsilon_{x;t}∼\text{Student}\prime \text{s}\;t(0,\sqrt{\frac{\nu_{x}-2}{\nu_{x}}},\nu_{x}\;). \end{array}$$ (4)

Now, by applying standard Student’s *t* distribution to GARCH(1,1) model, the conditional probability function of *X*_*t*_, given information $F_{x;t-1}$, is

$$\begin{array}{c} f_{X_{t}|F_{x;t-1}}\left(x\right)=\frac{1}{\sqrt{\sigma_{x;t}^{2}\left(\nu_{x}-2\right)}\;B\left(\frac{\nu_{x}}{2},\frac{1}{2}\right)}{\left[1+\frac{x^{2}}{\sigma_{x;t}^{2}\left(\nu_{x}-2\right)}\right]}^{-\frac{\nu_{x}+1}{2}},\;x\in R. \end{array}$$

In other words, $X_{t}|F_{x;t-1}$ is Student’s *t* distributed with parameter $\left(\nu_{x},\sigma_{x;t}\sqrt{\frac{\nu_{x}-2}{\nu_{x}}}\;\right)$. The explicit form of the conditional probability function above leads us to employ the conditional maximum likelihood method, as in McNeil et al. [4], to estimate its parameters. Note that this procedure is analogous to $Y_{t}|F_{y;t-1}$ and $Z_{t}|F_{z;t-1}$.

### Copula-based modeling

#### Copula and dependence measures

Suppose that a continuous bivariate random variable (*X*, *Y*), representing a joint risk, has marginal risk distribution function *F*_*X*_ and *F*_*Y*_, respectively. Suppose also that *U* = *F*_*X*_(*X*) and *V* = *F*_*Y*_(*Y*) so that they are uniformly distributed over unit interval, i.e. U,V∼U(0,1). Copula *C*_*U*,*V*_ is a joint distribution function of (*U*, *V*) such that

$$\begin{array}{c} C_{U,V}\left(u,v\right)=P\left(U\leq u,V\leq v\right),\;\left(u,v\right)\in {\left[0,1\right]}^{2}, \end{array}$$

where *C*_*U*,*V*_: [0, 1]^2^ → [0, 1]. Its corresponding probability function is

$$\begin{array}{c} c_{U,V}\left(u,v\right)=\frac{\partial^{2}C_{U,V}\left(u,v\right)}{\partial u\;\partial v}, \end{array}$$

which is called copula density, see e.g. Nelsen [36] and McNeil et al. [4]. Furthermore, Sklar’s theorem stated that joint distribution function *F*_*X*,*Y*_ of (*X*, *Y*) may be determined through copula *C*_*U*,*V*_ of marginal distribution functions *F*_*X*_ and *F*_*Y*_, i.e.

$$\begin{array}{c} F_{X,Y}\left(x,y\right)=C_{U,V}\left[F_{X}\left(x\right),F_{Y}\left(y\right)\right],\;\left(x,y\right)\in R^{2}. \end{array}$$

Sklar’s theorem has shown us that the use of copula provides many choices of joint distribution function of (*X*, *Y*). The corresponding joint probability function *f*_*X*,*Y*_ of (*X*, *Y*) is given by

$$\begin{array}{c} f_{X,Y}\left(x,y\right)=f_{X}\left(x\right)\cdot f_{Y}\left(y\right)\cdot c_{U,V}\left[F_{X}\left(x\right),F_{Y}\left(y\right)\right]. \end{array}$$

To measure dependence between *X* and *Y*, the dependence measures of Pearson’s *ρ* and Kendall’s *τ* are required. According to Schweizer and Wolff [37], such dependence measures are defined as

$$\begin{array}{cc} \rho_{X,Y} & =\rho \left(X,Y\right)=\frac{\iint_{R^{2}}\left[F_{X,Y}\left(x,y\right)-F_{X}\left(x\right)F_{Y}\left(y\right)\right]\;dx\;dy}{\sqrt{V(X)}\sqrt{V(Y)}},\\ \tau_{X,Y} & =\tau \left(X,Y\right)=4\iint_{R^{2}}F_{X,Y}\left(x,y\right)\;dF_{X,Y}\left(x,y\right)-1, \end{array}$$

respectively. By substituting *u* = *F*_*X*_(*x*) and *v* = *F*_*Y*_(*y*), they may be formulated as

$$\begin{array}{c} \rho_{X,Y}=\frac{\iint_{{\left[0,1\right]}^{2}}\left[C_{U,V}\left(u,v\right)-uv\right]\;dF_{X}^{-1}\left(u\right)\;dF_{Y}^{-1}\left(v\right)}{\sqrt{V(X)}\sqrt{V(Y)}} \end{array}$$ (5)

and

$$\begin{array}{c} \tau_{X,Y}=4\iint_{{\left[0,1\right]}^{2}}C_{U,V}\left(u,v\right)\;dC_{U,V}\left(u,v\right)-1=4\;E\left[C_{U,V}\left(U,V\right)\right]-1. \end{array}$$ (6)

It is shown from Eq (5) that Pearson’s *ρ*_*X*,*Y*_ depends not only on copula but also on marginal of *X* and *Y*. Thus, Pearson’s *ρ* is invariant only under linear transformation. Meanwhile, from Eq (6), it is shown that Kendall’s *τ*_*X*,*Y*_ depends only on copula. In addition, *τ*_*X*,*Y*_ = *τ*_*U*,*V*_. Thus, Kendall’s *τ* is invariant under both linear and nonlinear transformations. We may say that Kendall’s *τ* is copula-based dependence measure. Note that for parameter *θ* of copula *C*_*U*,*V*_, Kendall’s *τ*_*U*,*V*_ is a function of *θ* i.e. *τ*_*U*,*V*_(*θ*).

There are several copulas commonly used such as Archimedean and elliptical copulas. Based on Joe [38], examples of Archimedean copulas are Clayton, Gumbel and Frank with the following function and the corresponding density:

1. Clayton copula: $C_{U,V}^{\text{Clayton}}\left(u,v;\theta \right)={\left(u^{-\theta }+v^{-\theta }-1\right)}^{-\frac{1}{\theta }}$, where *θ* ∈ (0, ∞). Its corresponding copula density is
  $$\begin{array}{c} c_{U,V}^{\text{Clayton}}\left(u,v;\theta \right)=\left(1+\theta \right){\left(uv\right)}^{-1-\theta }{\left(u^{-\theta }+v^{-\theta }-1\right)}^{-\frac{1}{\theta }-2}. \end{array}$$
2. Gumbel copula: $C_{U,V}^{\text{Gumbel}}\left(u,v;\theta \right)=\exp\left\{-{\left[{\left(-\ln u\right)}^{\theta }+{\left(-\ln v\right)}^{\theta }\right]}^{\frac{1}{\theta }}\right\}$, where *θ* ∈ [1, ∞). Its corresponding copula density is
  $$\begin{array}{c} c_{U,V}^{\text{Gumbel}}\left(u,v;\theta \right)=\frac{{\left(-\ln u\right)}^{\theta -1}{\left(-\ln v\right)}^{\theta -1}g\left(u,v\right)}{uv\cdot C_{U,V}^{\text{Gumbel}}\left(u,v;\theta \right)} \end{array}$$
  where $g\left(u,v\right)={\left[{\left(-\ln u\right)}^{\theta }+{\left(-\ln v\right)}^{\theta }\right]}^{\frac{2}{\theta }-2}+\left(\theta -1\right){\left[{\left(-\ln u\right)}^{\theta }+{\left(-\ln v\right)}^{\theta }\right]}^{\frac{1}{\theta }-2}$.
3. Frank copula: $C_{U,V}^{\text{Frank}}\left(u,v;\theta \right)=-\frac{1}{\theta }\ln[1+\frac{\left(e^{-\theta u}-1\right)\left(e^{-\theta v}-1\right)}{e^{-\theta }-1}]$, where θ∈R∈\{0}. Its corresponding density is
  $$\begin{array}{c} c_{U,V}^{\text{Frank}}\left(u,v;\theta \right)=\frac{\theta e^{-\theta u}e^{-\theta v}\left(e^{-\theta u}-1\right)\left(e^{-\theta v}-1\right)}{{\left(e^{-\theta }-1\right)}^{2}}{\left[1+\frac{\left(e^{-\theta u}-1\right)\left(e^{-\theta v}-1\right)}{e^{-\theta }-1}\right]}^{-2}. \end{array}$$

Meanwhile, Gaussian and Student’s *t* copulas are examples of elliptical copulas. Their copula functions are

$$\begin{array}{c} C_{U,V}^{\text{Gaussian}}\left(u,v;\theta \right)=\Phi_{2}\left[\Phi^{-1}\left(u\right),\Phi^{-1}\left(v\right);\theta \right] \end{array}$$

and

$$\begin{array}{c} C_{U,V}^{\text{Student}\prime \text{s}\;t}\left(u,v;\nu ,\theta \right)=F_{T,T^{\prime }}\left[F_{T}^{-1}\left(u;\nu \right),F_{T}^{-1}\left(v;\nu \right);\nu ,\theta \right], \end{array}$$

respectively, where *θ* ∈ (−1, 1). Note that Φ(⋅) and Φ_2_(⋅, ⋅;*θ*) are distribution function of standard univariate normal and standard bivariate normal random variables, respectively. Furthermore, *F*_*T*,*T*′_(⋅, ⋅;*ν*, *θ*) is joint distribution function of identical Student’s *t* random variables (*T*, *T*′) with marginal distribution function *F*_*T*_(⋅;*ν*) and degrees of freedom *ν* ∈ (0, ∞). The corresponding densities of Gaussian and Student’s *t* copulas are, respectively, given by

$$\begin{array}{c} c_{U,V}^{\text{Gaussian}}\left(u,v;\theta \right)=\frac{1}{\sqrt{1-\theta^{2}}}\exp[-\frac{\theta^{2}\left(x^{2}+y^{2}\right)-2\theta xy}{2(1-\theta^{2})}], \end{array}$$

where *x* = Φ^−1^(*u*) and *y* = Φ^−1^(*v*), and

$$\begin{array}{c} c_{U,V}^{\text{Student}\prime \text{s}\;t}\left(u,v;\nu ,\theta \right)=\frac{1}{\pi f_{T}\left(x;\nu \right)f_{T}\left(y;\nu \right)\sqrt{1-\theta^{2}}}{\left[1+\frac{x^{2}+y^{2}-2\theta xy}{\nu (1-\theta^{2})}\right]}^{-\frac{\nu +2}{2}}, \end{array}$$

where $x=F_{T}^{-1}\left(u;\nu \right)$ and $y=F_{T}^{-1}\left(v;\nu \right)$.

The dependence measures of Kendall’s *τ* for Archimedean and elliptical copulas are provided in Table 1. It is shown that Kendall’s *τ* may be expressed in copula parameter *θ*. In addition, copula parameter *θ* may also be expressed in Kendall’s *τ*, except for Frank copula.

**Table 1:** Dependence measures of Kendall’s *τ* for Archimedean and elliptical copulas.

Copula	Kendall’s *τ*_*U*,*V*_(*θ*)	Parameter *θ*(*τ*_*U*,*V*_)
Clayton(*θ*)	$\frac{\theta }{\theta +2}$	$\frac{2\tau_{U,V}}{1-\tau_{U,V}}$
Gumbel(*θ*)	$1-\frac{1}{\theta }$	$\frac{1}{1-\tau_{U,V}}$
Frank(*θ*)	$1-\frac{4[1-D_{1}\left(\theta \right)]}{\theta }$	$\tau_{U,V}^{-1}\left(\theta \right)$
Gaussian(*θ*)	$\frac{2}{\pi }\arcsin\left(\theta \right)$	$\sin(\frac{\pi }{2}\tau_{U,V})$
Student’s *t*(*θ*, *ν*)	$\frac{2}{\pi }\arcsin\left(\theta \right)$	$\sin(\frac{\pi }{2}\tau_{U,V})$

Note: Kendall’s *τ* is expressed as function of copula parameter *θ* and such *θ* is expressed as function of Kendall’s *τ*. For Frank copula, its Kendall’s *τ* is depend on Debye function of $D_{1}\left(\theta \right)=\frac{1}{\theta }\int_{0}^{\theta }\frac{t}{e^{t}-1}\;dt$ whose inverse function has no explicit form.

**Fig 1:**
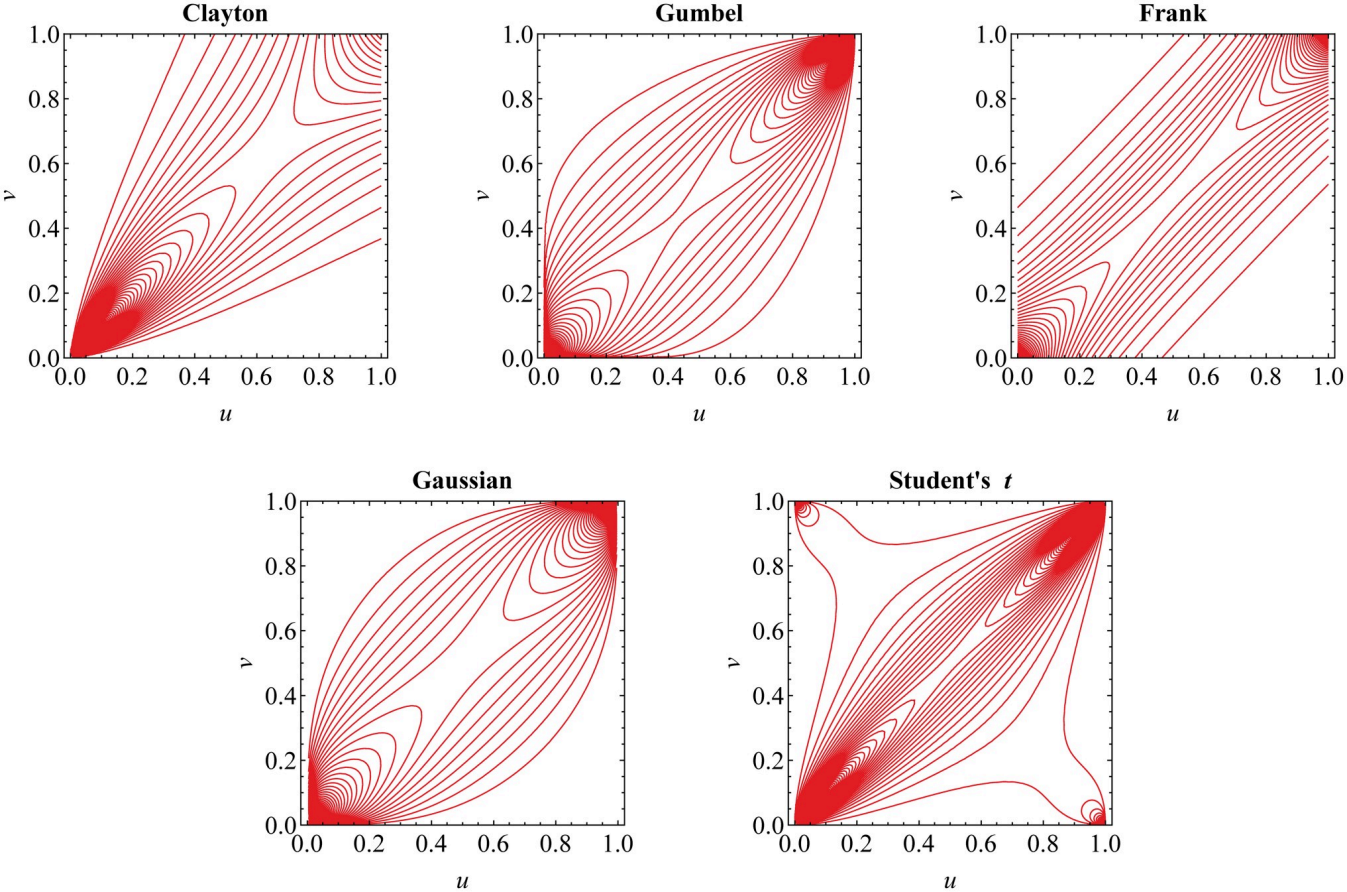
Contour plot for the density of Archimedean and elliptical copulas. The contours show their different dependence structure for equal Kendall’s *τ* = 0.6.

For equal value of Kendall’s *τ*, we present in Fig 1 the contour plot for the density of Archimedean and elliptical copulas. It may be observed that Clayton, Gumbel and Student’s *t* copulas perform tail dependence. In more detail, Clayton copula is appropriate for lower-tail dependent risks whilst upper-tail dependence may be captured by Gumbel copula. Furthermore, Student’s *t* copula displays symmetrical lower- and upper-tail dependence. Meanwhile, Frank and Gaussian copulas have symmetrical lower- and upper-tail independence.

#### Copula selection

For a given risk data set, the challenging task is selecting the best copula which fits well to the data. We may do this by considering several criteria. One of them is Akaike Information Criterion (AIC) introduced by Akaike [39]. Suppose that a copula *C*_*U*,*V*_(⋅, ⋅;**θ**) with its corresponding density *c*_*U*,*V*_(⋅, ⋅;**θ**) has parameter **θ**. Based on data ${\left\{\left(u_{i},v_{i}\right)\right\}}_{i=1}^{n}$ of (*U*, *V*), the estimate for such parameter may be obtained through maximum likelihood (ML) method with likelihood function $L\left(\theta \right)=\prod_{i=1}^{n}c_{U,V}\left(u_{i},v_{i};\theta \right)$. By replacing *θ* with its estimate, $\hat{\theta }$, we have ${\hat{C}}_{U,V}\left(\cdot ,\cdot ;\hat{\theta }\right)$ as the parametric estimate for *C*_*U*,*V*_(⋅, ⋅;**θ**). The AIC value from such copula is defined as

$$\begin{array}{c} \text{AIC}=-2\ln L(\hat{\theta })+2b, \end{array}$$

where *b* is number of parameters in **θ**. Among several choices of copulas, a copula with the lowest value of AIC may be decided as the best copula.

In addition, we employ other criteria by considering empirical version of copula. The empirical copula ${\hat{C}}_{n}$, for the data ${\left\{\left(u_{i},v_{i}\right)\right\}}_{i=1}^{n}$, is defined as

$$\begin{array}{c} {\hat{C}}_{n}\left(u,v\right)=\frac{1}{n}\sum_{i=1}^{n}I_{\left\{u_{i}\leq u,v_{i}\leq v\right\}},\;\left(u,v\right)\in {\left[0,1\right]}^{2}, \end{array}$$

where $I_{A}$ is indicator function on set *A*. Through graphical approach, the best fitting copula may typically be selected by comparing the surface of such empirical copula to that of ${\hat{C}}_{U,V}\left(u,v;\hat{\theta }\right)$. To give more convenient interpretation, we consider its univariate version i.e.

$$\begin{array}{c} {\hat{K}}_{n}\left(q\right)=\frac{1}{n}\sum_{j=1}^{n}I_{\left\{C_{n}\left(u_{j},v_{j}\right)\leq q\right\}},\;q\in \left[0,1\right]. \end{array}$$ (7)

Such empirical function is the nonparametric estimate of distribution function, K(q;θ)=P(Q≤q), for the so-called Kendall’s transform, *Q* = *C*_*U*,*V*_(*U*, *V*;*θ*), defining Kendall’s *τ* in Eq 6. According to Genest and Rivest [40], this leads us to visualize the curve for ${\hat{K}}_{n}\left(q\right)$ and its corresponding function of λ, defined by

$$\begin{array}{c} {\hat{\lambda }}_{n}\left(q\right)=q-{\hat{K}}_{n}\left(q\right), \end{array}$$ (8)

along with the parametric version derived from ${\hat{C}}_{U,V}\left(u,v;\hat{\theta }\right)$. Furthermore, we may perform a goodness-of-fit (GoF) test for such copula. It is carried out by testing

$$\begin{array}{c} {\text{H}}_{0}:C_{U,V}={\hat{C}}_{U,V}\left(\cdot ,\cdot ;\hat{\theta }\right)\;\text{versus}\;{\text{H}}_{1}:C_{U,V}\neq {\hat{C}}_{U,V}\left(\cdot ,\cdot ;\hat{\theta }\right), \end{array}$$

or, equivalently, testing ${\text{H}}_{0}:K=\hat{K}\left(\cdot ;\hat{\theta }\right)$ versus ${\text{H}}_{1}:K\neq \hat{K}\left(\cdot ;\hat{\theta }\right)$, where *C*_*U*,*V*_ and *K* are the true copula and the corresponding true Kendall’s distribution function, respectively. We use Cramér–von Mises test statistic

$$\begin{array}{c} S_{n}^{\left(K\right)}=\int_{0}^{1}n{\left[{\hat{K}}_{n}\left(q\right)-\hat{K}\left(q;\hat{\theta }\right)\right]}^{2}\;d{\hat{K}}_{n}\left(q\right) \end{array}$$

and calculate p-value of the test by adopting parametric bootstrap as proposed by Genest et al. [41].

### Vine copula-based modeling

#### Pair-copula construction method

Suppose that (*X*, *Y*, *Z*) is a dependent risk model consisting of three continuous random variables with joint probability function

$$\begin{array}{c} f_{X,Y,Z}=f_{X}\cdot f_{Y}\cdot f_{Z}\cdot c_{X,Y,Z}, \end{array}$$ (9)

where the marginal risk probability functions are *f*_*X*_, *f*_*Y*_, *f*_*Z*_ and *c*_*X*,*Y*, *Z*_ is a trivariate copula density. Since there is limitation on classes of trivariate copulas, we aim to find joint distribution of (*X*, *Y*, *Z*) through another approach. Joe [42] suggested a decomposition for *f*_*X*,*Y*, *Z*_ as

$$\begin{array}{c} f_{X,Y,Z}=f_{X}\cdot f_{Y|X}\cdot f_{Z|X,Y}. \end{array}$$

Note that

$$\begin{array}{c} f_{Y|X}=\frac{f_{X,Y}}{f_{X}}=\frac{f_{X}\cdot f_{Y}\cdot c_{X,Y}}{f_{X}}=f_{Y}\cdot c_{X,Y} \end{array}$$

and

$$\begin{array}{c} f_{Z|X,Y}=\frac{f_{Y,Z|X}}{f_{Y|X}}=\frac{f_{Y|X}\cdot f_{Z|X}\cdot c_{Y,Z|X}}{f_{Y|X}}=f_{Z|X}\cdot c_{Y,Z|X}. \end{array}$$

Since

$$\begin{array}{c} f_{Z|X}=\frac{f_{X,Z}}{f_{X}}=\frac{f_{X}\cdot f_{Z}\cdot c_{X,Z}}{f_{X}}=f_{Z}\cdot c_{X,Z}, \end{array}$$

we have *f*_*Z*|*X*, *Y*_ = *f*_*Z*_ ⋅ *c*_*X*,*Z*_ ⋅ *c*_*Y*,*Z*|*X*_. Thus,

$$\begin{array}{c} f_{X,Y,Z}=f_{X}\cdot f_{Y}\cdot f_{Z}\cdot c_{X,Y}\cdot c_{X,Z}\cdot c_{Y,Z|X}. \end{array}$$ (10)

Now, from Eqs (9) and (10), we obtain

$$\begin{array}{c} c_{X,Y,Z}=c_{X,Y}\cdot c_{X,Z}\cdot c_{Y,Z|X}. \end{array}$$ (11)

This shows that the trivariate copula density *c*_*X*,*Y*, *Z*_ for (*X*, *Y*, *Z*) model may be constructed from bivariate copula densities: *c*_*X*,*Y*_, *c*_*X*,*Z*_, *c*_*Y*,*Z*|*X*_. In other words, it provides flexible way to determine joint distribution of (*X*, *Y*, *Z*). The first two components *c*_*X*,*Y*_ and *c*_*X*,*Z*_ show dependence of (*X*, *Y*) and of (*X*, *Z*), respectively, with dependence measure *κ*_*X*,*Y*_ and *κ*_*X*,*Z*_. Meanwhile, the component *c*_*Y*,*Z*|*X*_ shows partial dependence of (*Y*, *Z*), given *X*, with partial dependence measure *κ*_*Y*,*Z*|*X*_. Note that the dependence measure of *κ* may be either Person’s *ρ* or Kendall’s *τ*.

Suppose that *F*_*X*_, *F*_*Y*_, *F*_*Z*_ denote marginal distribution functions of (*X*, *Y*, *Z*). The copula density *c*_*X*,*Y*, *Z*_ in Eq 11 is basically expressed as

$$\begin{array}{cc} c_{X,Y,Z}\left[F_{X}\left(x\right),F_{Y}\left(y\right),F_{Z}\left(z\right)\right] & =c_{X,Y}\left[F_{X}\left(x\right),F_{Y}\left(y\right)\right]\times c_{X,Z}\left[F_{X}\left(x\right),F_{Z}\left(z\right)\right]\\ & \times c_{Y,Z|X}\left[F_{Y|X}\left(y|x\right),F_{Z|X}\left(z|x\right)\right] \end{array}$$

for all $\left(x,y,z\right)\in R^{3}$, where *F*_*Y*|*X*_ and *F*_*Z*|*X*_ are conditional distribution function of *Y* and *Z*, given *X*, respectively. According to Joe [42], they may be defined as

$$\begin{array}{cc} F_{Y|X}\left(y|x\right) & =\frac{\partial C_{X,Y}\left[F_{X}\left(x\right),F_{Y}\left(y\right)\right]}{\partial F_{X}\left(x\right)}=C_{Y|X}\left[F_{Y}\left(y\right)|F_{X}\left(x\right)\right], \end{array}$$ (12)

$$\begin{array}{cc} F_{Z|X}\left(z|x\right) & =\frac{\partial C_{X,Z}\left[F_{X}\left(x\right),F_{Z}\left(z\right)\right]}{\partial F_{X}\left(x\right)}=C_{Z|X}\left[F_{Z}\left(z\right)|F_{X}\left(x\right)\right]. \end{array}$$ (13)

This shows that conditional copula density *c*_*Y*,*Z*|*X*_ is determined through copula *C*_*X*,*Y*_ and *C*_*Y*,*Z*_. From Eqs (12) and (13), copula density *c*_*X*,*Y*, *Z*_ is completely given by

$$\begin{array}{cc} c_{X,Y,Z}\left[F_{X}\left(x\right),F_{Y}\left(y\right),F_{Z}\left(z\right)\right] & =c_{X,Y}\left[F_{X}\left(x\right),F_{Y}\left(y\right)\right]\times c_{X,Z}\left[F_{X}\left(x\right),F_{Z}\left(z\right)\right]\\ & \times c_{Y,Z|X}\left\{C_{Y|X}\left[F_{Y}\left(y\right)|F_{X}\left(x\right)\right],C_{Z|X}\left[F_{Z}\left(z\right)|F_{X}\left(x\right)\right]\right\}. \end{array}$$

According to Joe [38], function of *C*_*Y*|*X*_ for each copula *C*_*X*,*Y*_, from Archimedean and elliptical copulas, is given in Table 2. Now, for *u* = *F*_*X*_(*x*), *v* = *F*_*Y*_(*y*) and *w* = *F*_*Z*_(*z*), copula density *c*_*X*,*Y*, *Z*_ is given by

$$\begin{array}{c} c_{X,Y,Z}\left(u,v,w\right)=c_{X,Y}\left(u,v\right)\cdot c_{X,Z}\left(u,w\right)\cdot c_{Y,Z|X}\left[C_{Y|X}\left(v|u\right),C_{Z|X}\left(w|u\right)\right] \end{array}$$

for all (*u*, *v*, *w*)∈[0, 1]^3^. Furthermore, if

$$\begin{array}{cc} v_{u} & =C_{Y|X}\left(v|u\right)=C_{Y|X}\left[F_{Y}\left(y\right)|F_{X}\left(x\right)\right],\\ w_{u} & =C_{Z|X}\left(w|u\right)=C_{Z|X}\left[F_{Z}\left(z\right)|F_{X}\left(x\right)\right], \end{array}$$

such copula density is as follows

$$\begin{array}{c} c_{X,Y,Z}\left(u,v,w\right)=c_{X,Y}\left(u,v\right)\cdot c_{X,Z}\left(u,w\right)\cdot c_{Y,Z|X}\left(v_{u},w_{u}\right). \end{array}$$

**Table 2:** Function of *C*_*Y*|*X*_.

Copula	Function of *C*_*Y*|*X*_(*v*|*u*)
Clayton	$u^{-\theta -1}{\left(u^{-\theta }+v^{-\theta }-1\right)}^{-\frac{1}{\theta }-1}$
Gumbel	$u^{-1}{\left(-\ln u\right)}^{\theta -1}{\left[{\left(-\ln u\right)}^{\theta }+{\left(-\ln v\right)}^{\theta }\right]}^{\frac{1}{\theta }-1}C_{X,Y}^{\text{Gumbel}}\left(u,v\right)$
Frank	e^−*θu*^[(e^−*θ*^ − 1)(e^−*θv*^ − 1)^−1^ + (e^−*θu*^ − 1)]^−1^
Gaussian	$\Phi [\frac{\Phi^{-1}\left(v\right)-\theta \Phi^{-1}\left(u\right)}{\sqrt{1-\theta^{2}}}]$
Student’s *t*	$F_{T}[\frac{F_{T}^{-1}\left(v;\nu \right)-\theta F_{T}^{-1}\left(u;\nu \right)}{\sqrt{\left(1-\theta^{2}\right)\left(\nu +{\left[F_{T}^{-1}\left(u;\nu \right)\right]}^{2}\right)/\left(\nu +1\right)}};\nu +1]$

Note: The function of *C*_*Y*|*X*_ is derived for Archimedean and elliptical copulas.

#### Graph structure and vine (copula)

Suppose that *V* denotes an empty and finite set. Suppose also that [*V*]^2^ = {{*v*, *v*′}:*v*, *v*′ ∈ *V*} is a set of all pairs of two unordered elements in *V*. According to Diestel [43], a graph is a pair (*V*, *E*) of sets for some *E* ⊆ [*V*]^2^. The element of *V* is called node whilst the element of *E* is an edge. A simple graph T=(V,E) that is connected and has no cycle is called a tree graph.

Based on Bedford and Cooke [44, 45] and Kurowicka and Cooke [46], $V_{m}$ is called vine on *m* elements, with $E\left(V_{m}\right)=E_{1}\cup E_{2}\cup \ldots \cup E_{m-1}$, if

1. $V_{m}=\left\{T_{1},T_{2},...,T_{m-1}\right\}$;
2. $T_{1}$ is a tree graph with a set of nodes *V*_1_ = {1, 2, …, *m*} and a set of (*m* − 1) edges *E*_1_ ⊆ [*V*_1_]^2^;
3. for all *j* = 2, 3, …, *m* − 1, $T_{j}$ is a tree graph with a set of nodes *V*_*j*_ = *E*_*j*−1_ and a set of edges *E*_*j*_ ⊆ [*V*_*j*_]^2^.

In short, vine is a collection of nested trees where edge of the *j*th tree is a node of the (*j* + 1)th tree.

**Fig 2:**
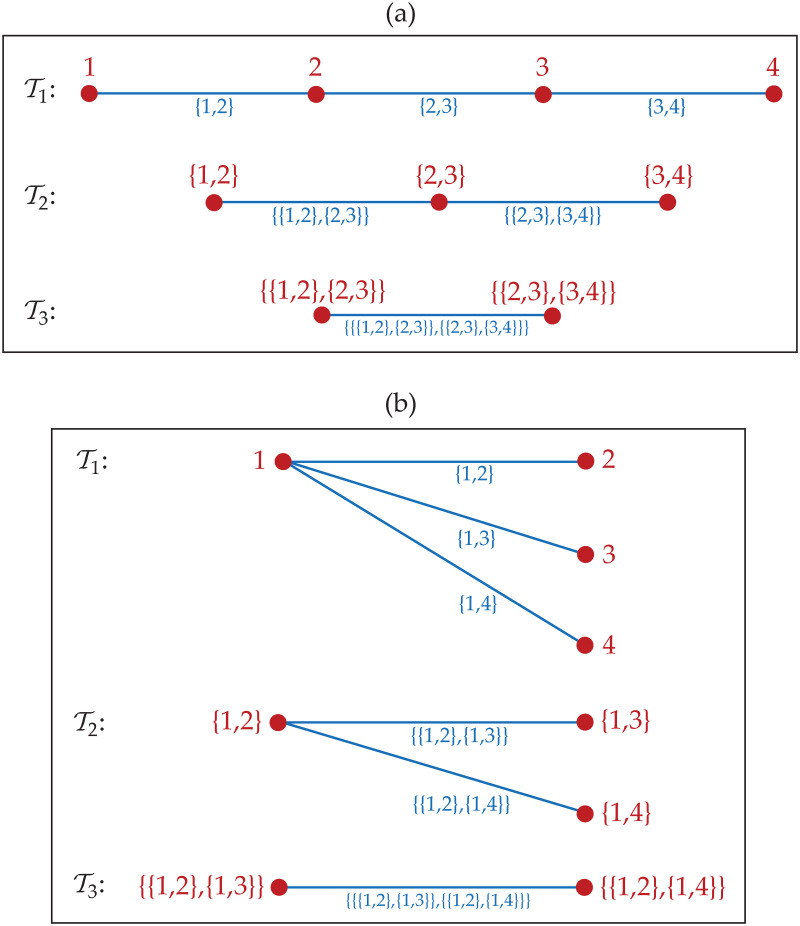
Graph structure of R-vine. The R-vine is $V_{4}=\left\{T_{1},T_{2},T_{3}\right\}$ as a (a) D-vine and as a (b) C-vine.

A vine $V_{m}$ is called a regular vine (R-vine) on *m* elements if “$V_{m}$ satisfies proximity condition, that is for all *j* = 2, 3, …, *m* − 1, if $v_{j},v_{j}^{\prime }\in V_{j}$ where $\left\{v_{j},v_{j}^{\prime }\right\}\in E_{j}$, then $|v_{j}\cap v_{j}^{\prime }|=1$. In other words, two adjacent nodes in the tree graph $T_{j}$ are two adjacent edges in the tree graph $T_{j-1}$”. There are two special cases of R-vine that are drawable vine (D-vine) and canonical vine (C-vine). R-vine $V_{m}$ is a D-vine if all nodes in the tree graph $T_{1}$ have maximum degrees of 2. Meanwhile, $V_{m}$ is called a C-vine if the tree graph $T_{j}$ has exactly one node with degrees of *m* − *j*, for *j* = 1, 2, …, *m* − 1; in tree graph $T_{1}$, such node is called a root. For an illustration, it is shown in Fig 2 graph structure of D-vine and C-vine, for *m* = 4 and $V_{4}=\left\{T_{1},T_{2},T_{3}\right\}$.

Now, an R-vine $V_{3}$ is employed to represent dependence model (*X*, *Y*, *Z*) determined through trivariate copula by pair-copula construction method. Here, node is a random variable whilst edge is a bivariate copula added by absolute dependence measure as weight. Such weight is used to find appropriate R-vine i.e. an R-vine where each tree graph has maximum of the sum of weights, according to Dißmann et al. [47].

First, define a complete graph $K_{3}$ (or a cricle graph $C_{3}$) with three nodes representing random variables of *X*, *Y*, *Z*. Let absolute value of each dependence measure *κ*_*X*,*Y*_, *κ*_*X*,*Z*_, *κ*_*Y*,*Z*_ be defined as weight of edge that connects two nodes. Then, select a maximum spanning tree graph, that is a tree subgraph of the complete graph $K_{3}$ maximizing the sum of weights. We denote the resulted R-vine copula by $\left(F,V_{3},C,K\right)$, where **F** is a collection of marginal distribution functions of (*X*, *Y*, *Z*). Meanwhile, **C** and **K** consist of bivariate copulas and the corresponding dependence measures, respectively.

### Forecasting Value-at-Risk (VaR)

#### Individual VaR forecast

The one-step-ahead VaR forecast is actually a forecast(ing limit) of future risk or return, *X*_*n*+1_, given previous information up to time *n*, $F_{x;n}$. At a specified confidence level of 1 − *α*, it may be calculated through the following formula, see e.g. McNeil et al. [4] and Syuhada [34],

$$\begin{array}{c} {\text{VaR}}_{x;n+1}^{1-\alpha }=\inf\left\{x:P\left(X_{n+1}\leq x|F_{x;n}\right)\geq 1-\alpha \right\}, \end{array}$$

for *α* ∈ (0, 1). Provided that the inverse of distribution function of *X*_*n*+1_, given $F_{x;n}$, exists, we may obtain ${\text{VaR}}_{x;n+1}^{1-\alpha }=F_{X_{n+1}|F_{x;n}}^{-1}\left(1-\alpha \right)$, according to Syuhada et al. [48]. This means that such VaR forecast is basically the (1 − *α*)-quantile of (conditional) distribution of the future return.

Since we use standard Student’s *t* for innovation distribution and assume GARCH(1,1) for (negative) return model, the conditional distribution of *X*_*n*+1_, given $F_{x;n}$, is Student’s *t* with degrees of freedom *ν*_*x*_ and scale parameter $\sigma_{x;n+1}\sqrt{\frac{\nu_{x}-2}{\nu_{x}}}$. This implies that its conditional mean and variance are

$$\begin{array}{c} E\left(X_{n+1}|F_{x;n}\right)=0,\;V\left(X_{n+1}|F_{x;n}\right)=\sigma_{x;n+1}^{2}=\omega_{x}+\delta_{x}\;x_{n}^{2}+\beta_{x}\;\sigma_{x;n}^{2}, \end{array}$$

respectively. Note that Student’s *t* is a symmetrical distribution. Thus, the one-step-ahead VaR forecast for *X*_*n*+1_, given $F_{x;n}$, consists of such conditional mean and variance, i.e. ${\text{VaR}}_{x;n+1}^{1-\alpha }=\sigma_{x;n+1}\;F_{\varepsilon_{x}}^{-1}\left(1-\alpha \right)$. Since $\varepsilon_{x;t}=T_{x}\sqrt{\frac{\nu_{x}-2}{\nu_{x}}}$, we obtain

$$\begin{array}{cc} {\text{VaR}}_{x;n+1}^{1-\alpha } & =\sigma_{x;n+1}\sqrt{\frac{\nu_{x}-2}{\nu_{x}}}\;F_{T_{x}}^{-1}\left(1-\alpha ;\nu_{x}\right)\\ & =\sqrt{\left(\omega_{x}+\delta_{x}\;x_{n}^{2}+\beta_{x}\;\sigma_{x;n}^{2}\right)\frac{\nu_{x}-2}{\nu_{x}}}\;F_{T_{x}}^{-1}\left(1-\alpha ;\nu_{x}\right). \end{array}$$

Parameter of model, (*ω*_*x*_, *δ*_*x*_, *β*_*x*_, *ν*_*x*_), may be replaced by its estimate so that we have the estimative one-step-ahead VaR forecast given by

$$\begin{array}{c} \hat{\text{VaR}}{}_{x;n+1}^{1-\alpha }=\sqrt{\left({\hat{\omega }}_{x}+{\hat{\delta }}_{x}\;x_{n}^{2}+{\hat{\beta }}_{x}\;{\hat{\sigma }}_{x;n}^{2}\right)\frac{{\hat{\nu }}_{x}-2}{{\hat{\nu }}_{x}}}\;F_{T_{x}}^{-1}\left(1-\alpha ;{\hat{\nu }}_{x}\right). \end{array}$$ (14)

In addition, the estimative *ℓ*(>1)-step-ahead VaR forecast is as follows

$$\begin{array}{c} \hat{\text{VaR}}{}_{x;n+\ell }^{1-\alpha }=\sqrt{\left({\hat{\omega }}_{x}+{\hat{\delta }}_{x}\;{\hat{X}}_{n+\ell -1}^{2}+{\hat{\beta }}_{x}\;\hat{\sigma }{}_{x;n+\ell -1}^{2}\right)\frac{{\hat{\nu }}_{x}-2}{{\hat{\nu }}_{x}}}\;F_{T_{x}}^{-1}\left(1-\alpha ;{\hat{\nu }}_{x}\right), \end{array}$$ (15)

where $\hat{\sigma }{}_{x;n+\ell -1}^{2}={\hat{\omega }}_{x}+{\hat{\delta }}_{x}\;{\hat{X}}_{n+\ell -1}^{2}+{\hat{\beta }}_{x}\;\hat{\sigma }{}_{x;n+\ell -2}^{2}$. Note that forecasting VaR for *Y*_*t*_, given $F_{y;t-1}$, and *Z*_*t*_, given $F_{z;t-1}$, may be carried out through the similar procedure.

#### Aggregate VaR forecast

For the case of a portfolio or aggregate risk, we aim to forecast VaR for future aggregate risk of

$$\begin{array}{c} S_{n+1}=X_{n+1}+Y_{n+1}+Z_{n+1}, \end{array}$$

given previous information $F_{x,y,z;n}$. We may employ vine copula to determine the joint distribution of model $(X_{n+1}^{*},Y_{n+1}^{*},Z_{n+1}^{*})=$ $\left(X_{n+1}|F_{x;n},Y_{n+1}|F_{y;n},Z_{n+1}|F_{z;n}\right)$. Suppose that $f_{X_{n+1}^{*},Y_{n+1}^{*},Z_{n+1}^{*}}$ denotes its joint probability function having a certain decomposition. Then, the conditional distribution function of *S*_*n*+1_, given $F_{x,y,z;n}$, is determined as follows

$$\begin{array}{cc} F_{S_{n+1}|F_{x,y,z;n}}\left(s\right) & =P(S_{n+1}\leq s|F_{x,y,z;n})\\ & =P(X_{n+1}+Y_{n+1}+Z_{n+1}\leq s|F_{x,y,z;n})\\ & =\int_{-\infty }^{s}\int_{-\infty }^{s-x}\int_{-\infty }^{s-x-y}f_{X_{n+1}^{*},Y_{n+1}^{*},Z_{n+1}^{*}}\left(x,y,z\right)\;dz\;dy\;dx,\;s\in R. \end{array}$$

By employing vine copula, forecasting the one-step-ahead individual VaRs is simultaneously carried out based on the joint distribution of $\left(X_{n+1}^{*},Y_{n+1}^{*},Z_{n+1}^{*}\right)$. We collect all of them into a vector denoted by

$$\begin{array}{c} V_{n+1}={\left({\text{VaR}}_{x;n+1}^{1-\alpha },{\text{VaR}}_{y;n+1}^{1-\alpha },{\text{VaR}}_{z;n+1}^{1-\alpha }\right)}^{T}. \end{array}$$ (16)

Then, we may forecast the one-step-ahead aggregate VaR, ${\text{aggVaR}}_{s;n+1}^{1-\alpha }$, by taking such individual VaR forecasts into account. We do this by first observing that

$$\begin{array}{c} {\text{aggVaR}}_{s;n+1}^{1-\alpha }={\left(V_{n+1}^{T}PV_{n+1}\right)}^{1/2}, \end{array}$$ (17)

where

$$\begin{array}{c} P=(\begin{array}{ccc} 1 & \kappa_{x,y} & \kappa_{x,z}\\ \kappa_{x,y} & 1 & \kappa_{y,z}\\ \kappa_{x,z} & \kappa_{y,z} & 1 \end{array}) \end{array}$$

is matrix of dependence measure for $\left(X_{n+1}^{*},Y_{n+1}^{*},Z_{n+1}^{*}\right)$. From Eq (17), through simple algebraic operation we have

$$\begin{array}{cc} {\text{aggVaR}}_{s;n+1}^{1-\alpha } & =[{\left({\text{VaR}}_{x;n+1}^{1-\alpha }\right)}^{2}+{\left({\text{VaR}}_{y;n+1}^{1-\alpha }\right)}^{2}+{\left({\text{VaR}}_{z;n+1}^{1-\alpha }\right)}^{2}+2\kappa_{x,y}\left({\text{VaR}}_{x;n+1}^{1-\alpha }\right)\left({\text{VaR}}_{y;n+1}^{1-\alpha }\right)\\ & +2\kappa_{x,z}\left({\text{VaR}}_{x;n+1}^{1-\alpha }\right)\left({\text{VaR}}_{z;n+1}^{1-\alpha }\right)+2\kappa_{y,z}\left({\text{VaR}}_{y;n+1}^{1-\alpha }\right)\left({\text{VaR}}_{z;n+1}^{1-\alpha }\right){]}^{1/2}. \end{array}$$

This means that the aggVaR forecast above incorporates interactions between different returns by introducing their dependence measures. Note that the estimative aggVaR forecast may be obtained by using the individual estimative VaR forecasts and the estimate of dependence measures.

For perfect (positive) dependence, i.e. *κ*_*x*,*y*_ = *κ*_*x*,*z*_ = *κ*_*y*,*z*_ = 1, it is obvious that the aggVaR forecast is equal to the simple sum of individual VaR forecasts written by

$$\begin{array}{c} {\text{simplesumVaR}}_{s;n+1}^{1-\alpha }={\text{VaR}}_{x;n+1}^{1-\alpha }+{\text{VaR}}_{y;n+1}^{1-\alpha }+{\text{VaR}}_{z;n+1}^{1-\alpha }. \end{array}$$ (18)

Thus, simplesumVaR may simply be decided as the aggVaR forecast when the worse cases of all of our risks always occur simultaneously, see e.g. Li et at. [49] and Embrechts et al. [2]. In other words, the risks have dependence representation of the form *M*(*u*, *v*, *w*) = min(*u*, *v*, *w*) which may be called perfect-dependence copula. However, since the dependence measures satisfy |*κ*_*x*,*y*_|, |*κ*_*x*,*z*_|, |*κ*_*y*,*z*_|≤1, we have

$$\begin{array}{c} {\text{aggVaR}}_{s;n+1}^{1-\alpha }\leq {\text{simplesumVaR}}_{s;n+1}^{1-\alpha }. \end{array}$$

This means that the aggVaR forecast in Eq (17) is bounded from above by simplesumVaR in Eq (18). The weaker the dependence among marginal risks, the lower the aggVaR forecast. This is inline with the fact that *M*(*u*, *v*, *w*) is the upper bound for all classes of copulas, see e.g. Joe [38], including pair-copula which determines the vine copula-based dependence among marginal risks for our portfolio risk.

In addition, it is interesting to investigate diversification benefits of using aggVaR and simplesumVaR. As in Li et at. [49], such diversification benefits may be measured by a so-called diversification coefficient (DC) defined as

$$\begin{array}{c} {\text{DC}}_{n+1}^{1-\alpha }=\frac{{\text{simplesumVaR}}_{s;n+1}^{1-\alpha }-{\text{aggVaR}}_{s;n+1}^{1-\alpha }}{{\text{simplesumVaR}}_{s;n+1}^{1-\alpha }}. \end{array}$$ (19)

From Eq (19), it may be understood that such coefficient is getting higher as the dependence among marginal risks is weaker. This makes the portfolio better diversified.

To find the *ℓ*-step-ahead aggregate VaR forecast, we consider the vector of ℓ-step-ahead individual VaR forecasts derived simultaneously. We denote it by

$$\begin{array}{c} V_{n+\ell }={\left({\text{VaR}}_{x;n+\ell }^{1-\alpha },{\text{VaR}}_{y;n+\ell }^{1-\alpha },{\text{VaR}}_{z;n+\ell }^{1-\alpha }\right)}^{T}; \end{array}$$ (20)

each component is obtained similarly by Eq (15). Then, we have

$$\begin{array}{c} {\text{aggVaR}}_{s;n+\ell }^{1-\alpha }={\left(V_{n+\ell }^{T}\;PV_{n+\ell }\right)}^{1/2}. \end{array}$$ (21)

The calculation of DC for this aggVaR forecast is analogous to Eq (19) by involving the simple sum of individual VaR forecasts of Eq (20).

### Backtesting VaR

After we find the (aggregate) VaR forecast, the assessment of the forecast accuracy is required. It may be carried out through backtesting. From a variety of the backtesting methods, we adopt the methods from Syuhada [34] and Jiménez et al. [20]. We discuss the following methods for the case of ${\text{VaR}}_{x;n+1}^{1-\alpha }$; the discussion for the other individual VaR and the aggregate VaR forecasts are similar.

#### Probability-based backtesting

As stated before, the one-step-ahead VaR forecast is the forecasting limit of future risk, given information of risks in the past. To simply asses the accuracy of VaR forecast, we, therefore, may calculate the probability that the actual future risks do not violate the VaR forecast. It is called coverage probability (CP) defined by

$$\begin{array}{c} \text{CP}=P(X_{n+1}\leq \hat{\text{VaR}}{}_{x;n+1}^{1-\alpha }|F_{x;n}). \end{array}$$

The closer the CP to the confidence level, 1 − *α*, the more accurate the VaR forecast.

When the sequence of actual future risks, ${\left\{x_{n+1;k}\right\}}_{k=1}^{N}$, is obtained, we may define ${\left\{I_{n+1;k}\right\}}_{k=1}^{N}$, where $I_{n+1;k}=I_{\left\{x_{n+1;k}\leq \hat{\text{VaR}}{}_{x;n+1}^{1-\alpha }\right\}}$, for *k* = 1, 2, …, *N*. We expect that ${\left\{I_{n+1;k}\right\}}_{k=1}^{N}$ is the sequence of realizations of Bernoulli random variable with parameter 1 − *α*. Thus, the value of $\frac{1}{N}\sum_{k=1}^{N}I_{n+1;k}$ is required to estimate the CP.

From the sequence of ${\left\{x_{n+1;k}\right\}}_{k=1}^{N}$, we may also require the sequence of ${\left\{I_{n+1;k}^{*}\right\}}_{k=1}^{N}$, where $I_{n+1;k}^{*}=I_{\left\{x_{n+1;k}>\hat{\text{VaR}}{}_{x;n+1}^{1-\alpha }\right\}}$, for *k* = 1, 2, …, *N*. Note that the term 1 refers to failure or violation of the VaR forecast; all terms are expected to be the realizations of Bernoulli random variable with failure proportion equal to *α*. Comparison of the actual number of failures, $\sum_{k=1}^{N}I_{n+1;k}^{*}$, and the expected number of failures, *Nα*, may be considered to assess the accuracy of VaR forecast. Their ratio is called actual over expected (AE) ratio.

#### Conditional coverage test

The standard test for assessing the accuracy of VaR forecast is the test whether the failure proportion is equal to *α*. It is basically carried out through binomial test by considering normal approximation of binomial distribution. However, according to Christoffersen [50], the forecast accuracy may be assessed by determining whether the sequence of ${\left\{I_{n+1;k}^{*}\right\}}_{k=1}^{N}$ consists of independent and identical realizations with failure proportion equal to *α*. This means that we need to combine the test of proportion of failure (PoF) and the test whether this sequence satisfies the conditional coverage independence (CCI). Their combination is called conditional coverage (CC) test.

The PoF and CCI tests employ likelihood ratio (LR) test statistic. The LR statistic for PoF test is defined by

$$\begin{array}{c} {\text{LR}}_{\text{PoF}}=-2\ln[{\left(\frac{1-\alpha }{1-\hat{\alpha }}\right)}^{N-N_{\text{f}}}{\left(\frac{\alpha }{\hat{\alpha }}\right)}^{N_{\text{f}}}], \end{array}$$

where $N_{\text{f}}=\sum_{k=1}^{N}I_{n+1;k}^{*}$ is the actual number of failures and $\hat{\alpha }=\frac{N_{\text{f}}}{N}$ is its actual proportion. Meanwhile, the LR statistic for CCI test is

$$\begin{array}{c} {\text{LR}}_{\text{CCI}}=-2\ln[\frac{{\left(1-\pi_{1}\right)}^{N_{00}+N_{10}}\;\pi_{1}^{N_{01}+N_{11}}}{{\left(1-P_{01}\right)}^{N_{00}}\;P_{01}^{N_{01}}\;{\left(1-P_{11}\right)}^{N_{10}}\;P_{11}^{N_{11}}}], \end{array}$$

where *N*_*ij*_ is number of the term *i* followed by the term *j* and *P*_*ij*_ is the corresponding transition probability, for *i*, *j* = 0, 1, from ${\left\{I_{n+1;k}^{*}\right\}}_{k=1}^{N}$ viewed as the sequence of realizations of a Markov chain model with binary states; *π*_1_ is unconditional probability of the term 1 (failure). Both LR statistics are asymptotically chi-square distributed with 1 degree of freedom. As proposed by Christoffersen [50], the CC test employs the LR statistic defined by LR_CC_ = LR_PoF_ + LR_CCI_; it is asymptotically chi-square distributed with 2 degrees of freedom. The null hypothesis of correct model specification fails to be rejected when p-value derived from this statistic is above significance level.

#### Backtesting through loss function

It is known that VaR is basically the quantile of (conditional) distribution of the future risk. In addition to the use of inverse of its distribution function, VaR as quantile may be formulated through different approach. Based on Kuan et al. [51], ${\text{VaR}}_{x;n+1}^{1-\alpha }$ is the minimizer of the loss function defined by

$$\begin{array}{c} L\left(a\right)=E[|\left(1-\alpha \right)-I_{\left\{X_{n+1}\leq a\right\}}\left|\cdot \right|X_{n+1}-a|,\;\text{given}\;F_{x;n}]. \end{array}$$

In this case, the loss function evaluated at our VaR forecast may be relatively compared to that evaluated at VaR forecast obtained from other method as benchmark. They are called quantile losses. The value of their ratio below one means that our forecasting method outperforms the benchmark.

## Result and discussion

### Daily returns, innovation distribution and marginal risks

**Fig 3:**
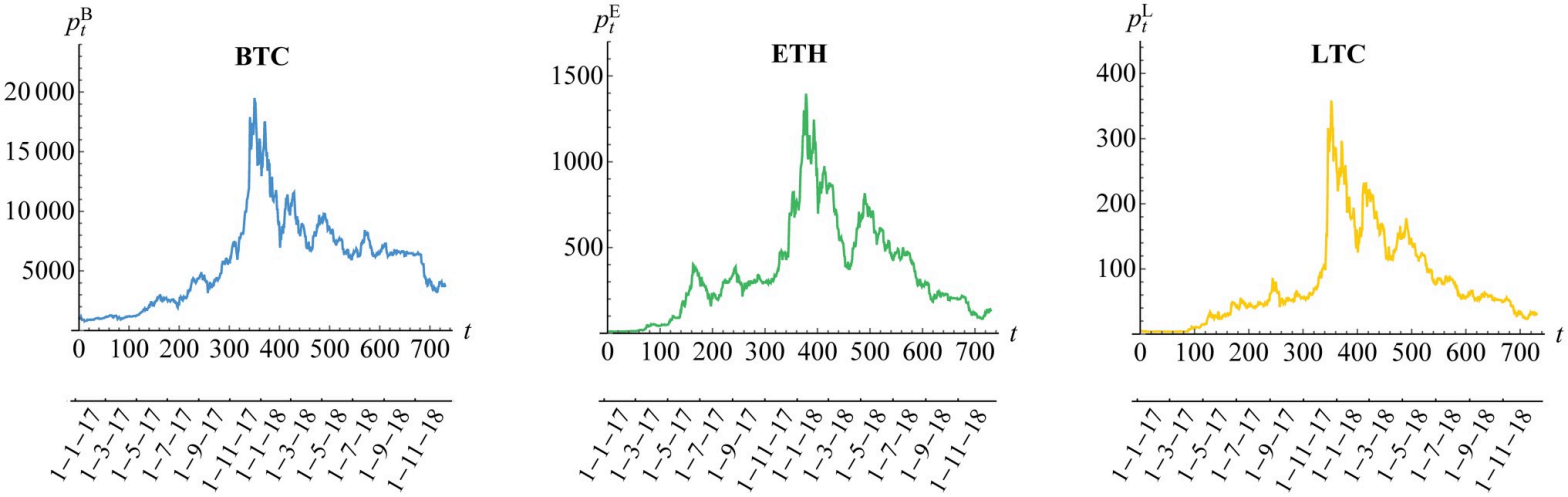
Daily closing prices of cryptocurrencies. The cryptocurrecy prices are $\left\{P_{t}^{B}\right\}$ for Bitcoin (BTC), $\left\{P_{t}^{E}\right\}$ for Ethereum (ETH) and $\left\{P_{t}^{L}\right\}$ for Litecoin (LTC).

**Fig 4:**
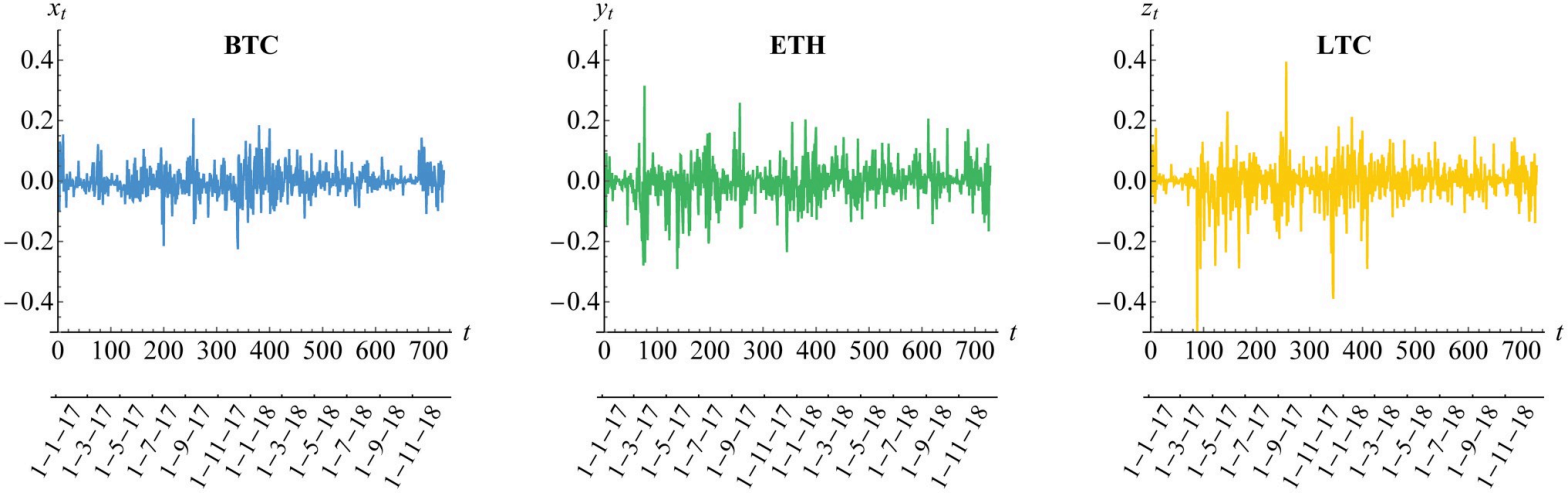
Negative returns of cryptocurrencies. The notations of such returns are {*X*_*t*_}, {*Y*_*t*_} and {*Z*_*t*_} for BTC, ETH and LTC, respectively.

**Fig 5:**
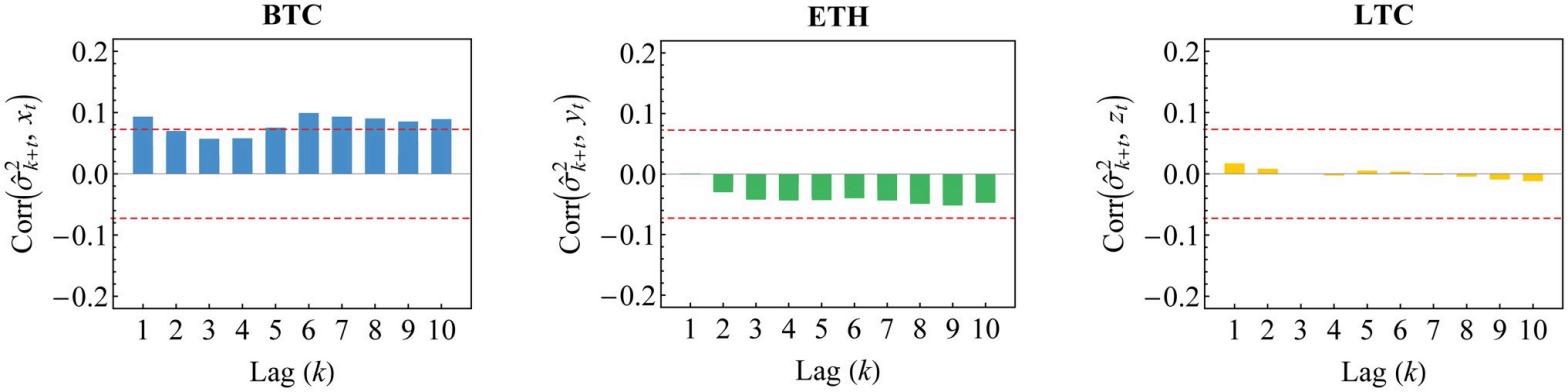
Correlation between (negative) return and its squared volatility. The significant positive correlation indicates (inverse) leverage effect in the returns data.

The dynamic daily prices and returns for Bitcoin (BTC), Ethereum (ETH) and Litecoin (LTC) for a period of 730 days in 2017-2018 are given in Figs 3 and 4, respectively. The returns follow formula in Eq (1). Whilst the three cryptocurrencies seem to have similar prices behavior, Litecoin has a dramatic increase in day 350 (December 2017) before the decrease in day 400 (February 2018), in comparison to Bitcoin (less dramatic) and Ethereum (gradual increase). In addition, the returns of Litecoin have more high volatility compared to the low and medium volatility as shown in Bitcoin and Ethereum returns, respectively. The property of dynamic volatility significantly appears in all of these returns based on the result of hypothesis test for ARCH effect of Engle [52] in Table 3. This result leads us to assume conditional heteroscedasticity for each of our risk models. Furthermore, the stationarity assumption is also needed based on the result of ADF test in Table 3. In addition, the existence of (inverse) leverage effect may also be considered, especially for Bitcoin whose (negative) return is positively correlated with its squared volatility as visualized in Fig 5. In other words, we also need to assume asymmetric heteroscedastic model in order to capture the feature of asymmetrical volatility. This is in line with assumption in Bouri et al. [25] and Klein et al. [26].

**Table 3:** Summary of test statistics for returns of Bitcoin (BTC), Ethereum (ETH) and Litecoin (LTC).

Test Statistic	BTC	ETH	LTC
ARCH PQ	105.3 (0.0000)	120.0 (0.0000)	48.7 (0.0000)
ADF	-717.5 (0.0000)	-709.1 (0.0000)	-717.7 (0.0000)

Note: The Portmanteau-Q (PQ) test statistic is used to test Engle’s ARCH effect or conditional heteroscedasticity whilst the augmented Dickey–Fuller (ADF) test is for stationarity. The null hypothesis of the tests is rejected due to low p-value (in parentheses) below 5% significance level.

We have assumed a GARCH(1,1), as in Eq (2), for each of our risk models. It consists of dynamic volatility and innovation. Table 4 gives the summary of statistics for the estimates of such innovation, defined in Eq (3), for the returns of Bitcoin, Ethereum and Litecoin. It is observed that the high empirical kurtosis leads us to employ heavy-tailed distribution for innovation.

**Table 4:** Summary of statistics for innovation estimates of Bitcoin (BTC), Ethereum (ETH) and Litecoin (LTC).

Statistic	BTC	ETH	LTC
Minimum	-16.8157	-4.3824	-7.9936
Maximum	4.6060	4.2687	4.9401
Mean	-0.0724	-0.0615	-0.0404
Median	-0.0745	-0.0076	0.0229
Std. Deviation	1.1679	0.9342	0.9326
Kurtosis	61.2525	6.2055	13.987

Note: The high kurtosis (above 3) indicates that the innovation is leptokurtic or heavy-tailed.

**Fig 6:**
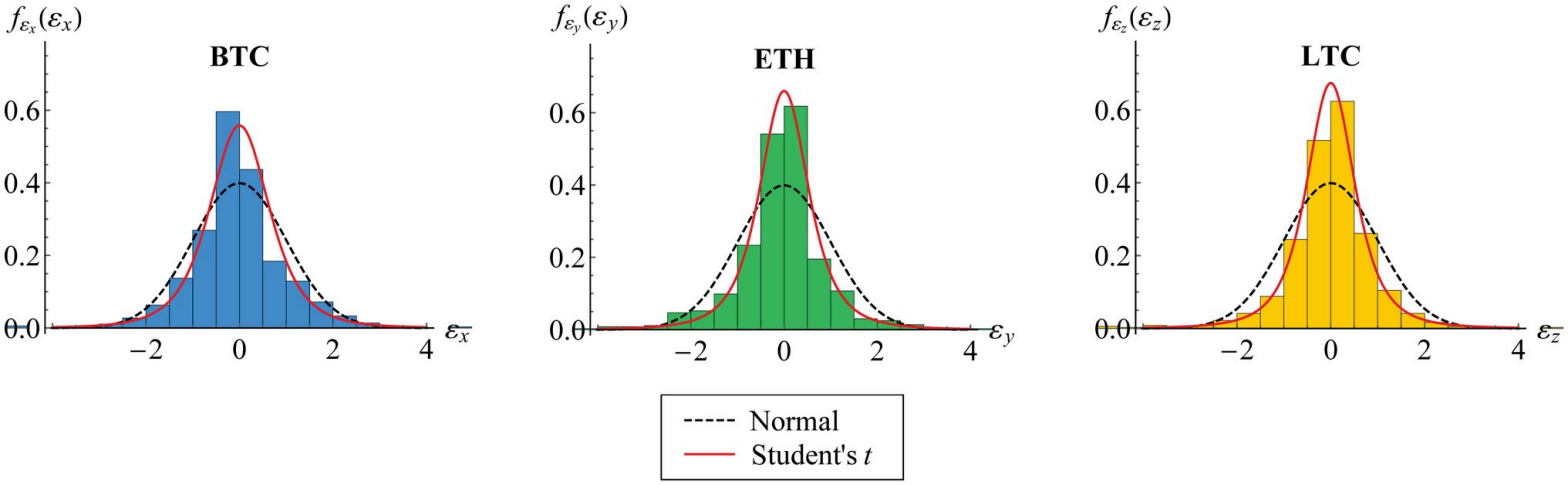
Histogram of innovation estimates. The histogram for BTC (blue), ETH (green) and LTC (yellow) is fitted to standard normal (black dashed) and standard Student’s *t* (red) probability functions.

**Fig 7:**
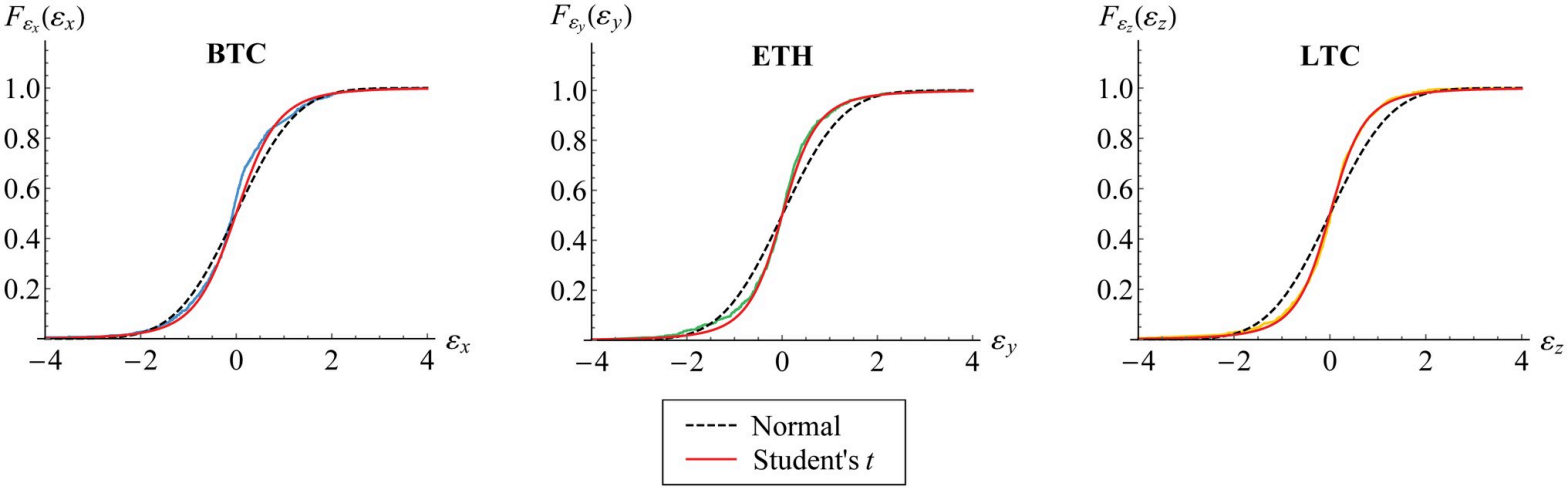
Empirical distribution function of innovations. The distribution function for BTC (blue), ETH (green) and LTC (yellow) is fitted to standard normal (black dashed) and standard Student’s *t* (red) distribution functions.

Furthermore, we visualize the innovation estimates in histogram, Fig 6. Both standard normal and standard Student’s *t* curves for probability function are fitted, where the estimated degrees of freedom of standard Student’s *t* are given in Table 5. In fact, it may be observed that standard normal distribution is not appropriate for each innovation. This is also described in Fig 7 for its distribution function. Based on AIC values also given in Table 5, standard Student’s *t* distribution has lower value of AIC than standard normal distribution. This confirms the appropriateness of standard Student’s *t* distribution for such innovation, as in Eq (4).

**Table 5:** The estimate for degrees of freedom of standard Student’s *t* distribution along with AIC value.

	BTC	ETH	LTC
Estimate for degrees of freedom	3.6111	2.8916	2.8363
AIC for standard Student’s *t*	**1988.1323**	**1823.1346**	**1745.1279**
AIC for standard normal	2335.3883	1977.2438	1973.5137

Note: The estimate for degrees of freedom is calculated through maximum likelihood method. Lower value of AIC is in boldface.

Based on the assumption above, parameter estimates of GARCH(1,1) model are given in Table 6. By assuming symmetrical volatility, it is observed that the persistence parameters ${\hat{\delta }}_{x}+{\hat{\beta }}_{x}$ (0.9256), ${\hat{\delta }}_{y}+{\hat{\beta }}_{y}$ (0.8178), ${\hat{\delta }}_{z}+{\hat{\beta }}_{z}$ (0.9239) are close to one but still lower than one. These also show stationarity of all processes {*X*_*t*_}, {*Y*_*t*_} and {*Z*_*t*_}. Meanwhile, for asymmetric model with considering inverse leverage effect, we need an additional parameter *γ* to ensure that the positive-signed returns lead a rise in the volatility. In other words, such volatility is modeled by

$$\begin{array}{c} \sigma_{x;t}^{2}=\omega_{x}+\delta_{x}\;X_{t-1}^{2}+\beta_{x}\;\sigma_{x;t-1}^{2}+\gamma_{x}\;X_{t-1}^{2}\;I_{\left\{X_{t-1}>0\right\}}, \end{array}$$

for (instance) Bitcoin returns. From Table 6, the stationarity condition is also satisfied for all asymmetric processes since the persistence parameters $\hat{\delta }+\hat{\beta }+\frac{1}{2}\hat{\gamma }=0.9247,0.8207,0.9239$ are below one. For Litecoin, we obtain ${\hat{\gamma }}_{z}=0$ which means that asymmetric GARCH(1,1) is equal to symmetric GARCH(1,1).

**Table 6:** The estimate for parameters of (a)symmetric GARCH(1,1) model.

Model	Parameter	BTC	ETH	LTC
Symmetric	*ω*	1.9285 × 10^−5^	18.432 2 × 10^−5^	3.1725× 10^−5^
	*δ*	0.0919	0.1202	0.0758
	*β*	0.8337	0.6976	0.8481
Asymmetric	*ω*	1.9955 × 10^−5^	18.0567 × 10^−5^	3.1725 × 10^−5^
	*δ*	0.0892	0.1105	0.0758
	*β*	0.8320	0.6995	0.8481
	*γ*	0.0070	0.0214	0

Note: The estimation is carried out through conditional maximum likelihood method.

### Dependence among innovations and the best copula selection

Modeling dependence of (*ε*_*x*;*t*_, *ε*_*y*;*t*_, *ε*_*z*;*t*_) is used to compute and to find joint distribution of $\left(X_{t}^{*},Y_{t}^{*},Z_{t}^{*}\right)=(X_{t}|F_{x;t-1},Y_{t}|F_{y;t-1},Z_{t}\left|F_{z;t-1}\right)$. As stated before, suppose that $\varepsilon_{x}=\left\{{\hat{\varepsilon }}_{x;t}\right\}$, $\varepsilon_{y}=\left\{{\hat{\varepsilon }}_{y;t}\right\}$, and $\varepsilon_{z}=\left\{{\hat{\varepsilon }}_{z;t}\right\}$. We calculate the dependence measure for each pair of innovation estimates and also calculate maximum of the sum of absolute dependence measures. Then, the appropriate trivariate copula density $c_{\varepsilon_{x;t},\varepsilon_{y;t},\varepsilon_{z;t}}$ to determine joint distribution of (*ε*_*x*;*t*_, *ε*_*y*;*t*_, *ε*_*z*;*t*_) is constructed.

**Fig 8:**
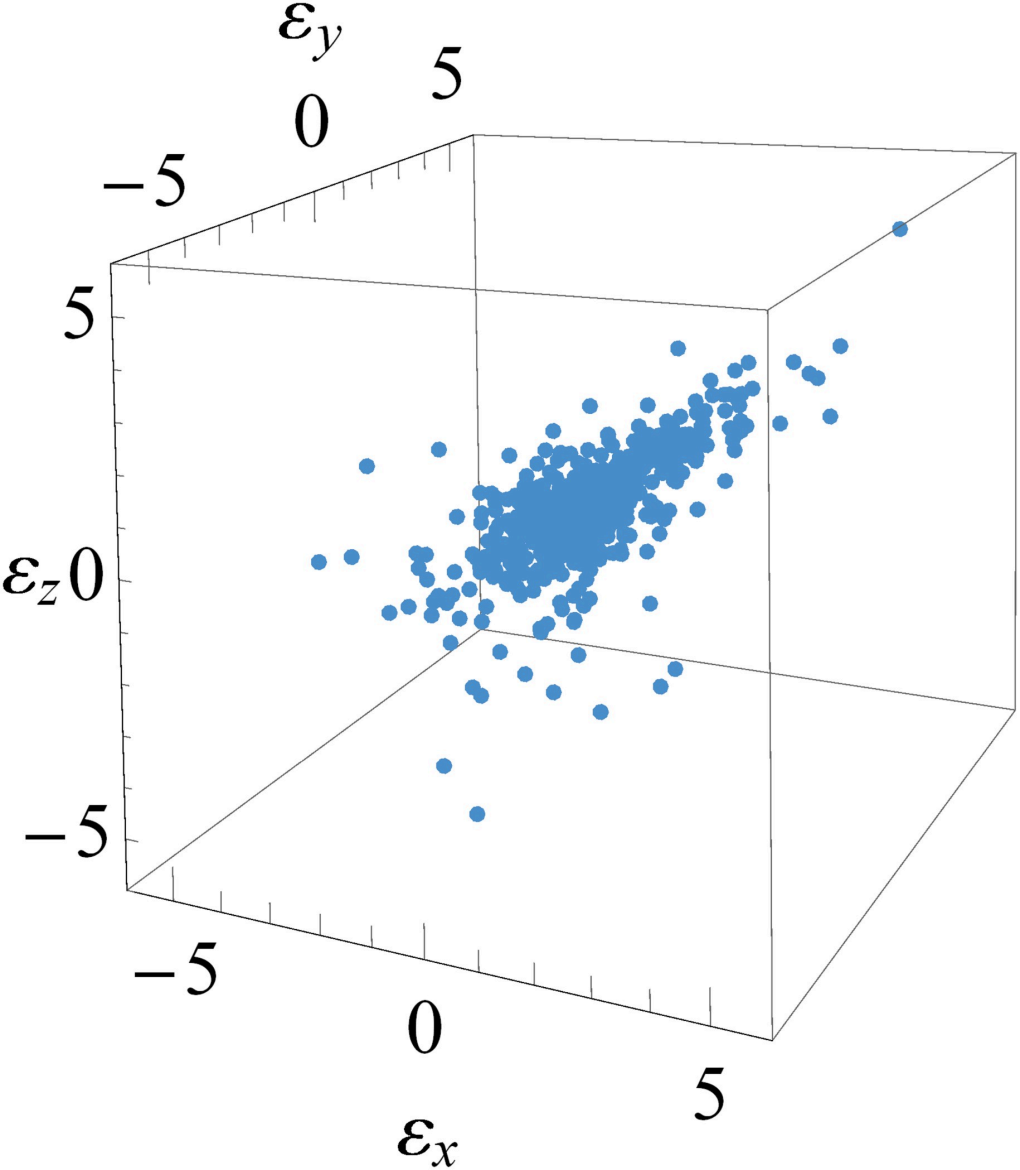
Three dimensional scatter plot of innovation estimates. The innovation estimates are $\left\{\left({\hat{\varepsilon }}_{x;t},{\hat{\varepsilon }}_{y;t},{\hat{\varepsilon }}_{z;t}\right)\right\}$.

**Fig 9:**
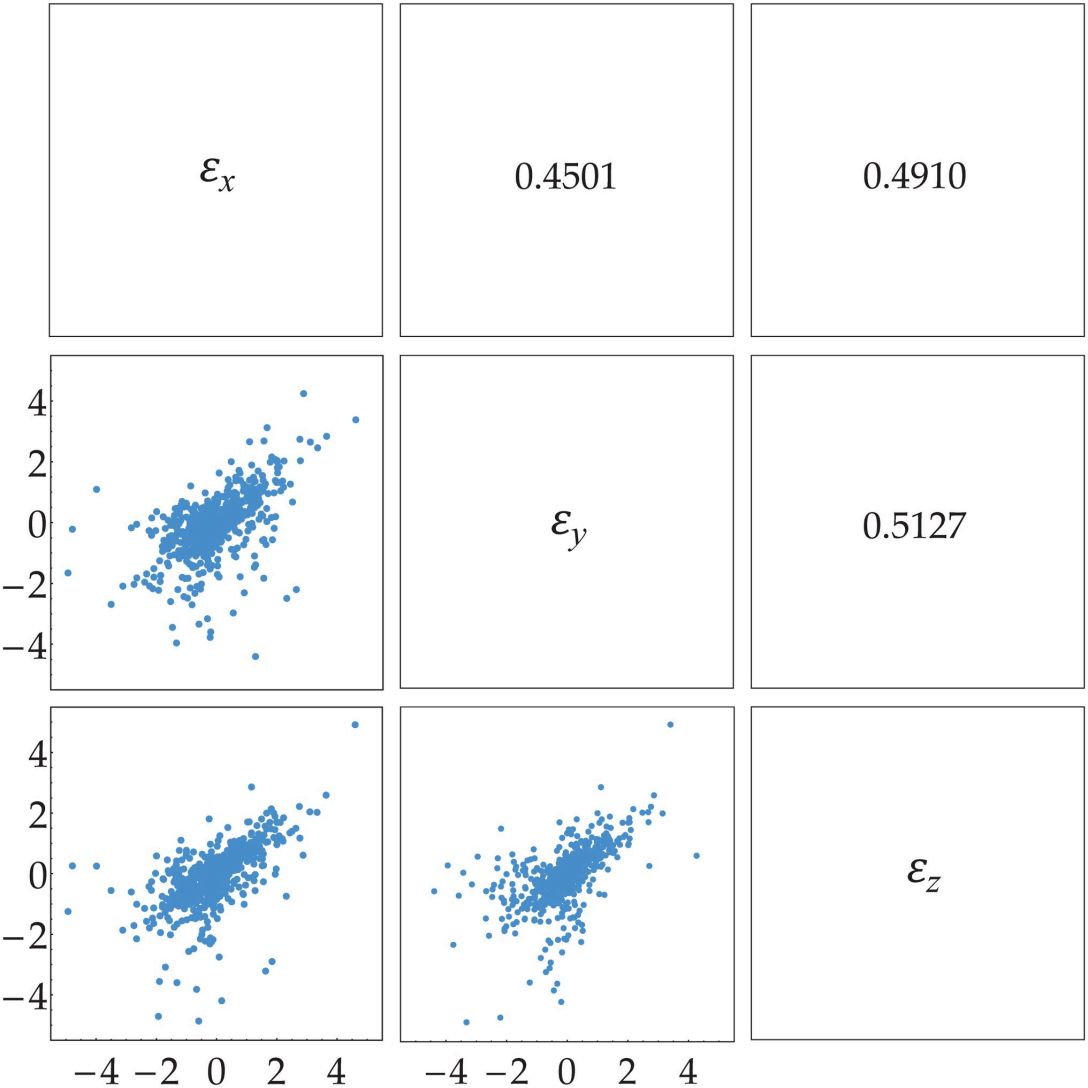
Two dimensional scatter plot of innovation estimates. The innovation estimates are $\left\{\left({\hat{\varepsilon }}_{x;t},{\hat{\varepsilon }}_{y;t}\right)\right\}$, $\left\{\left({\hat{\varepsilon }}_{x;t},{\hat{\varepsilon }}_{z;t}\right)\right\}$ and $\left\{\left({\hat{\varepsilon }}_{y;t},{\hat{\varepsilon }}_{z;t}\right)\right\}$ with the corresponding dependence measure ${\hat{\kappa }}_{x,y}=0.4501$, ${\hat{\kappa }}_{x,z}=0.4910$ and ${\hat{\kappa }}_{y,z}=0.5127$, respectively.

Fig 8 shows scatter plot (three dimension) of innovation estimates $\left\{\left({\hat{\varepsilon }}_{x;t},{\hat{\varepsilon }}_{y;t},{\hat{\varepsilon }}_{z;t}\right)\right\}$ whilst Fig 9 describes scatter plot of each two dimensional pair of innovation estimates: $\left\{\left({\hat{\varepsilon }}_{x;t},{\hat{\varepsilon }}_{y;t}\right)\right\}$, $\left\{\left({\hat{\varepsilon }}_{x;t},{\hat{\varepsilon }}_{z;t}\right)\right\}$ and $\left\{\left({\hat{\varepsilon }}_{y;t},{\hat{\varepsilon }}_{z;t}\right)\right\}$. Note that their dependence measures are ${\hat{\kappa }}_{x,y}=0.4501$, ${\hat{\kappa }}_{x,z}=0.4910$ and ${\hat{\kappa }}_{y,z}=0.5127$, respectively. This means that innovation estimates with maximum of the sum of absolute dependence measures are $\left\{\left({\hat{\varepsilon }}_{x;t},{\hat{\varepsilon }}_{z;t}\right)\right\}$ and $\left\{\left({\hat{\varepsilon }}_{y;t},{\hat{\varepsilon }}_{z;t}\right)\right\}$. Thus, the appropriate trivariate copula density $c_{\varepsilon_{x;t},\varepsilon_{y;t},\varepsilon_{z;t}}$ to determine joint distribution of (*ε*_*x*;*t*_, *ε*_*y*;*t*_, *ε*_*z*;*t*_) is

$$\begin{array}{c} c_{\varepsilon_{x;t},\varepsilon_{y;t},\varepsilon_{z;t}}=c_{\varepsilon_{x;t},\varepsilon_{z;t}}\cdot c_{\varepsilon_{y;t},\varepsilon_{z;t}}\cdot c_{\varepsilon_{x;t},\varepsilon_{y;t}|\varepsilon_{z;t}}. \end{array}$$

Note that the first two bivariate copula densities, respectively, correspond to bivariate copula $C_{\varepsilon_{x;t},\varepsilon_{z;t}}$ and $C_{\varepsilon_{y;t},\varepsilon_{z;t}}$ for edges of the tree graph $T_{1}$ whilst the last one corresponds to conditional copula $C_{\varepsilon_{x;t},\varepsilon_{y;t}|\varepsilon_{z;t}}$ for edge of the next tree graph $T_{2}$. The latter copula is defined through copula $C_{\varepsilon_{x;t},\varepsilon_{z;t}}$ and $C_{\varepsilon_{y;t},\varepsilon_{z;t}}$.

According to Aas et al. [11] and Czado et al. [53], copula parameters are estimated through sequential maximum likelihood method as follows. First, the innovation estimates ${\hat{\varepsilon }}_{x;t},{\hat{\varepsilon }}_{y;t},{\hat{\varepsilon }}_{z;t}$ are transformed through marginal distribution function $F_{\varepsilon_{x;t}},F_{\varepsilon_{y;t}},F_{\varepsilon_{z;t}}$ that are

$$\begin{array}{c} u_{t}=F_{\varepsilon_{x;t}}\left({\hat{\varepsilon }}_{x;t}\right),v_{t}=F_{\varepsilon_{y;t}}\left({\hat{\varepsilon }}_{y;t}\right),w_{t}=F_{\varepsilon_{z;t}}\left({\hat{\varepsilon }}_{z;t}\right)\in \left[0,1\right]. \end{array}$$

The transformed data are then used to estimate parameter of copula $C_{\varepsilon_{x;t},\varepsilon_{z;t}}\left(\cdot ,\cdot ;\theta_{x,z}\right)$ and $C_{\varepsilon_{y;t},\varepsilon_{z;t}}\left(\cdot ,\cdot ;\theta_{y,z}\right)$ from several classes. The best copulas for $C_{\varepsilon_{x;t},\varepsilon_{z;t}}$ and $C_{\varepsilon_{y;t},\varepsilon_{z;t}}$, based on several criteria, are used to find function $C_{\varepsilon_{x;t}|\varepsilon_{z;t}}$ and $C_{\varepsilon_{y;t}|\varepsilon_{z;t}}$, respectively. Now, define

$$\begin{array}{c} u_{w;t}={\hat{C}}_{\varepsilon_{x;t}|\varepsilon_{z;t}}\left(u_{t}|w_{t};{\hat{\theta }}_{x,z}\right),v_{w;t}={\hat{C}}_{\varepsilon_{y;t}|\varepsilon_{z;t}}\left(v_{t}|w_{t};{\hat{\theta }}_{y,z}\right)\in \left[0,1\right], \end{array}$$

so that we have the transformed data {(*u*_*w*;*t*_, *v*_*w*;*t*_)}. We then use these data to estimate parameter of copula $C_{\varepsilon_{x;t},\varepsilon_{y;t}|\varepsilon_{z;t}}\left(\cdot ,\cdot ;\theta_{x,y|z}\right)$.

**Fig 10:**
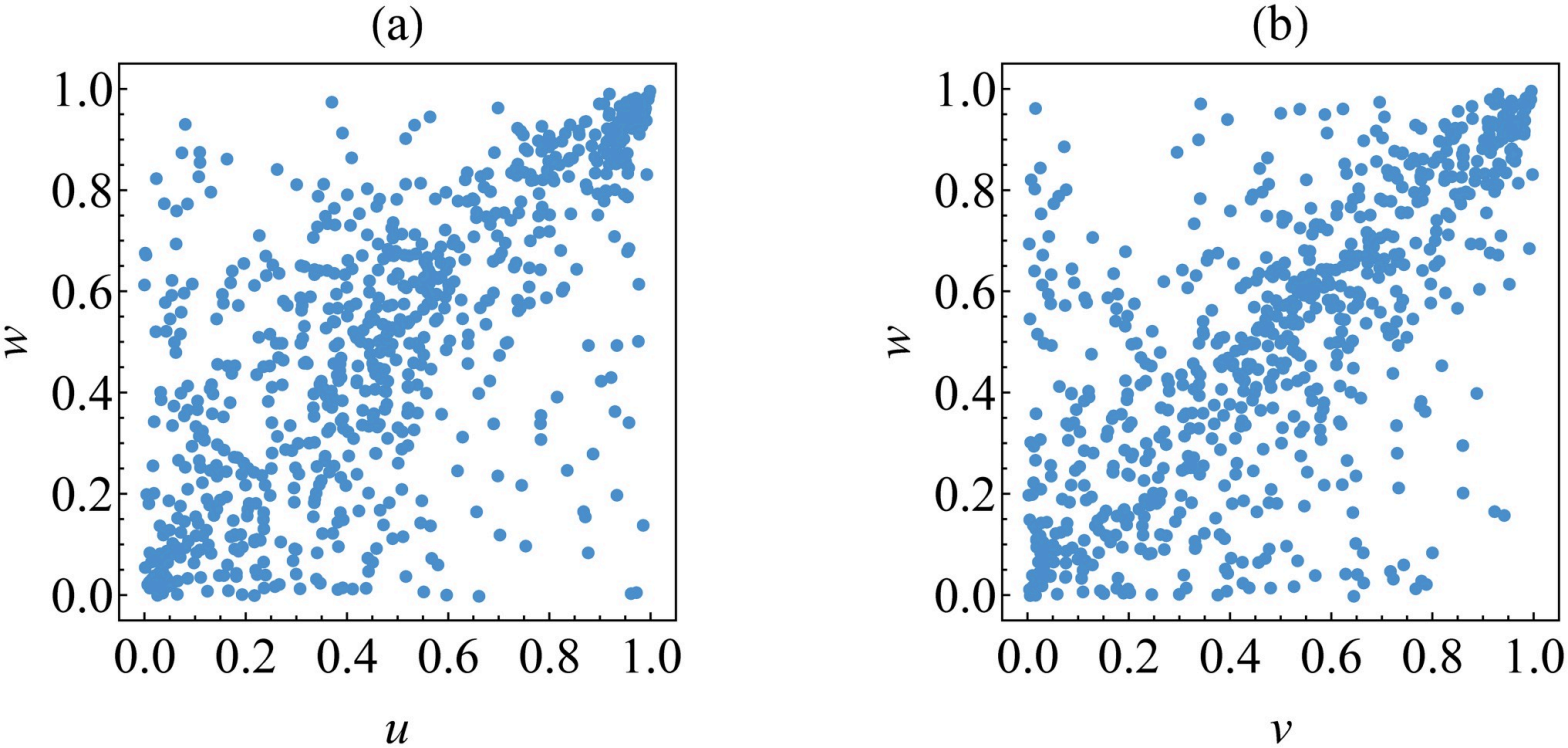
Transformed data for determining the best copulas for the first tree graph. The data are (a) {(*u*_*t*_, *w*_*t*_)} and (b) {(*v*_*t*_, *w*_*t*_)}.

The scatter plot of the transformed data {(*u*_*t*_, *w*_*t*_)} and {(*v*_*t*_, *w*_*t*_)} is shown in Fig 10. It may be observed that these transformed data of the estimated innovations show asymmetrical dependence with strong dependence in upper tails. This means that an increase of extremely positive innovation for BTC (or ETH) returns is followed by an increase of that for LTC returns. This empirical fact indicates that Gumbel copula is appropriate for such data. This indication is inline with the result of copula selection based on AIC and goodness-of-fit criteria provided in Table 7. This is because Gumbel copula has the lowest value of AIC and the highest p-value (>0.05) of the goodness-of-fit test for both data {(*u*_*t*_, *w*_*t*_)} and {(*v*_*t*_, *w*_*t*_)}.

**Table 7:** Parameter estimates along with the best copula selection for the first tree graph based on AIC and goodness-of-fit test by using data (a) {(*u*_*t*_, *w*_*t*_)} and (b) {(*v*_*t*_, *w*_*t*_)}.

Data	Copula	Estimate (Std. Error)	AIC	GoF (p-value)
(a)	Clayton	0.8202 (0.0680)	-208.3876	2.3732 (0.0000)
	Gumbel	1.9709 (0.0610)	**-513.3633**	0.0757 (**0.2445**)
	Frank	5.7798 (0.2920)	-440.1642	0.5330 (0.0000)
	Gaussian	0.6244 (0.0190)	-377.3082	0.3657 (0.0000)
	Student’s *t*	0.6894 (0.0210)	-484.0637	0.4091 (0.0000)
		3.0795 (0.4210)		
(b)	Clayton	0.7725 (0.0660)	-204.8118	3.4415 (0.0000)
	Gumbel	2.0281 (0.0630)	**-531.6440**	0.1133 (**0.0769**)
	Frank	6.1585 (0.3040)	-473.6364	0.4293 (0.0000)
	Gaussian	0.6339 (0.0180)	-389.5267	0.6240 (0.0000)
	Student’s *t*	0.7049 (0.0200)	-486.3833	0.5506 (0.0000)
		3.0055 (0.3880)		

Note: The estimation for parameter of Archimedean and elliptical copulas is carried out through maximum likelihood method whilst the goodness-of-fit test uses Cramér–von Mises statistic. The lowest value of AIC and the highest p-value for certain data are in boldface.

**Fig 11:**
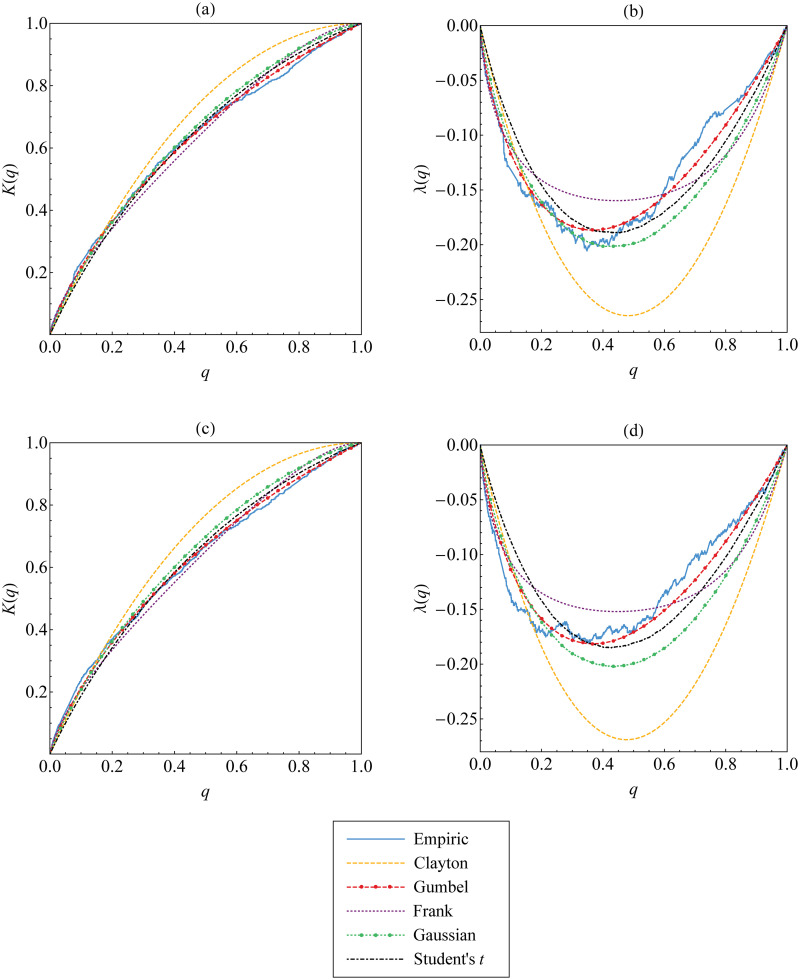
The best copula selection for the first tree graph based on Kendall’s distribution function and the corresponding function of λ. Such functions for Archimedean and elliptical copulas are compared and fitted to those for empirical copula of the data (a-b) {(*u*_*t*_, *w*_*t*_)} and (c-d) {(*v*_*t*_, *w*_*t*_)}.

In addition, graphical approach visualized in Fig 11 explains that Kendall’s distribution function (and the corresponding function of λ) derived from Gumbel copula seem to be close enough to the empirical Kendall’s distribution function defined in Eq (7) (and the empirical function of λ_*n*_ defined in Eq (8)). This means that Gumbel copula fits well to the upper-tail dependent data {(*u*_*t*_, *w*_*t*_)} and {(*v*_*t*_, *w*_*t*_)}.

**Fig 12:**
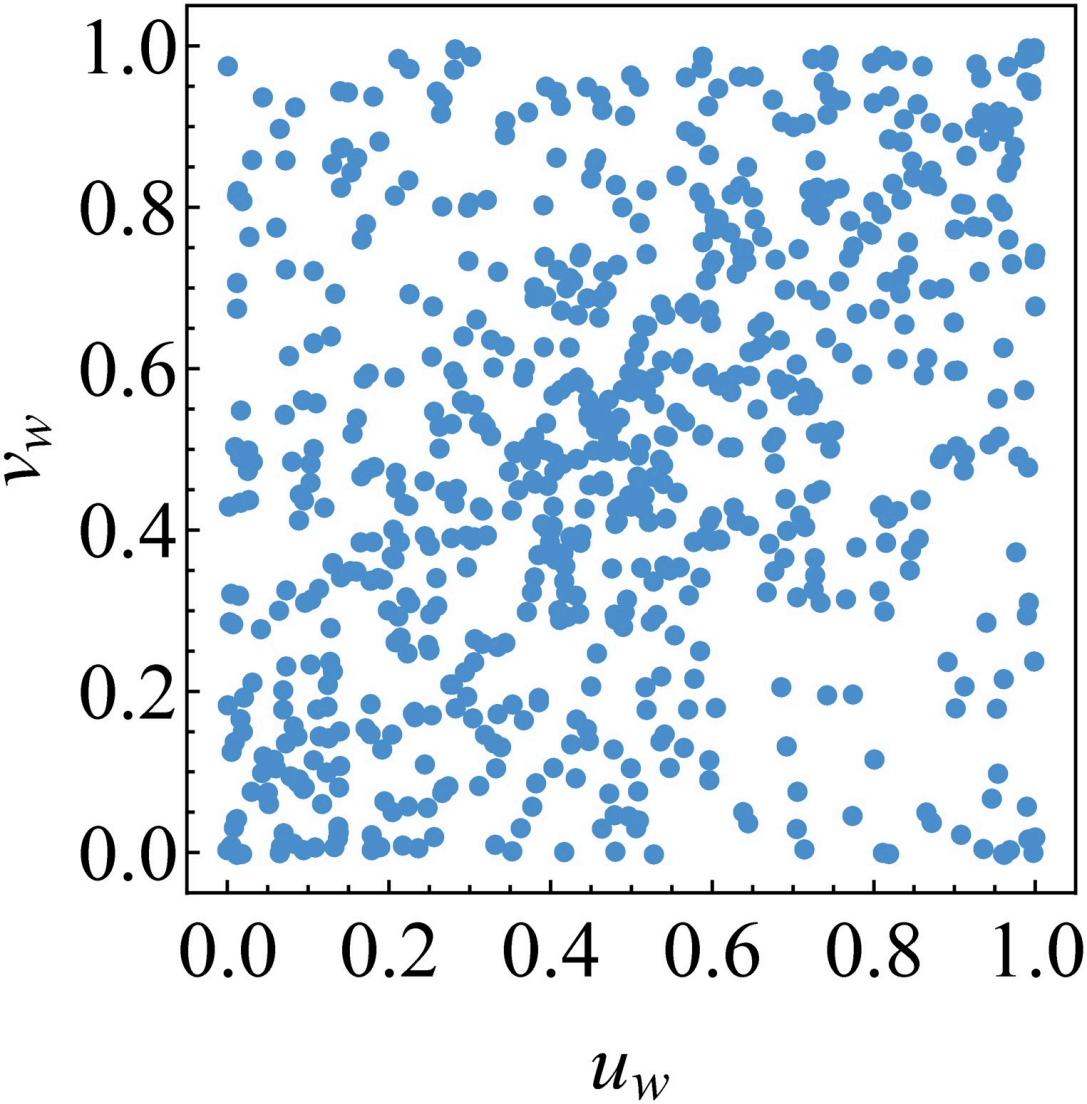
Transformed data for determining the best copula for the second tree graph. The data are {(*u*_*w*;*t*_, *v*_*w*;*t*_)} with ${\hat{\kappa }}_{u,v|w}=0.2301$.

**Fig 13:**
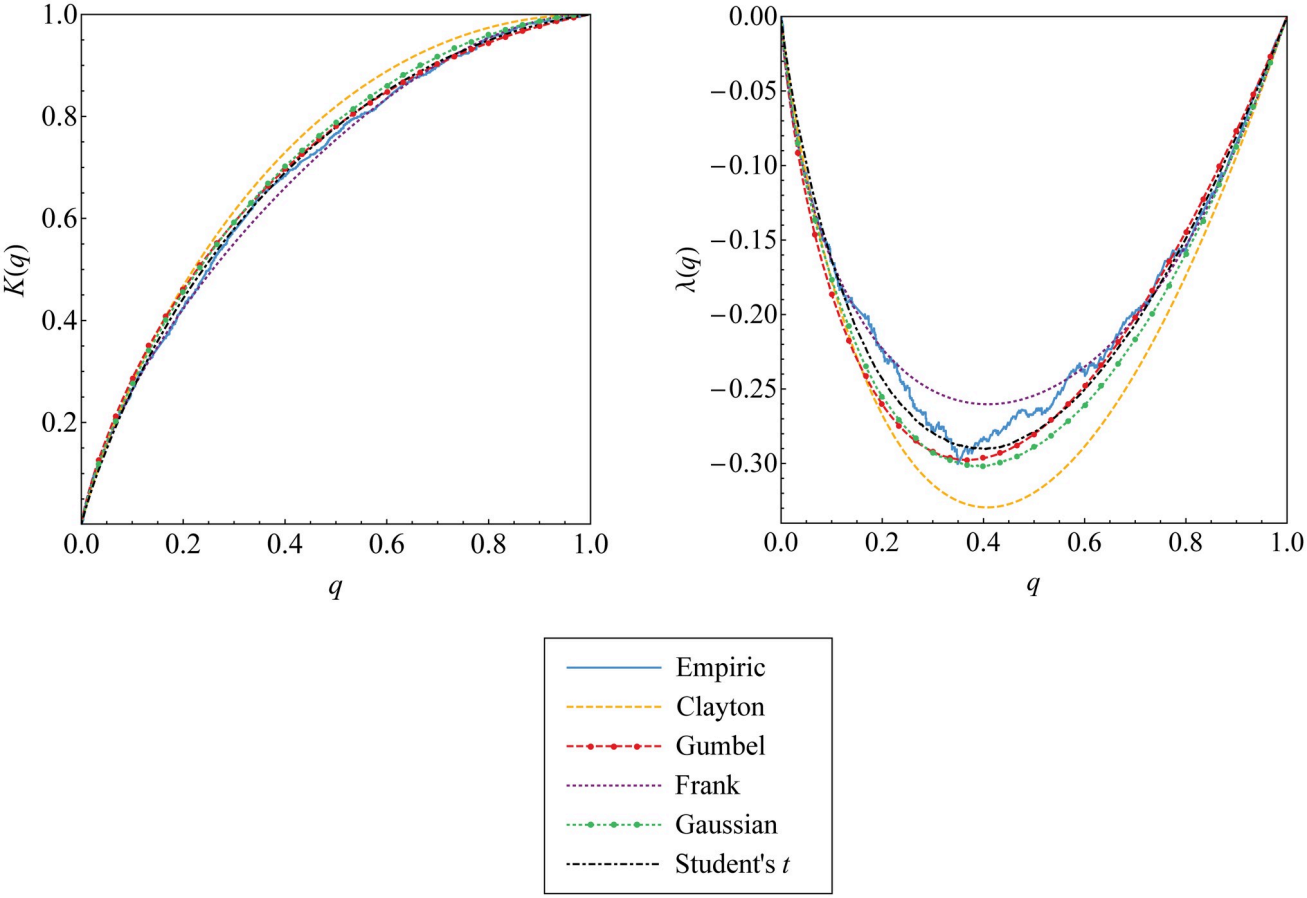
The best copula selection for the second tree graph based on Kendall’s distribution function and the corresponding function of λ. Such functions for Archimedean and elliptical copulas are compared and fitted to those for empirical copula of the data {(*u*_*w*;*t*_, *v*_*w*;*t*_)}.

From the result above, Gumbel copula is the best fitting copula for $C_{\varepsilon_{x;t},\varepsilon_{z;t}}$ and $C_{\varepsilon_{y;t},\varepsilon_{z;t}}$. By employing Gumbel copula for such copulas, we obtain the transformed data {(*u*_*w*;*t*_, *v*_*w*;*t*_)} shown in Fig 12. We now observe that the new transformed data show symmetrical tail dependence. This empirical fact leads us to consider Student’s *t* copula as the best fitting copula for $C_{\varepsilon_{x;t},\varepsilon_{y;t}|\varepsilon_{z;t}}$. This consideration is inline with the result of the best copula selection based on AIC and goodness-of-fit criteria, see Table 8, as well as graphical approach, see Fig 13.

**Table 8:** Parameter estimates along with the best copula selection for the second tree graph based on AIC and goodness-of-fit test by using data {(*u*_*w*;*t*_, *v*_*w*;*t*_)}.

Copula	Estimate (Std. Error)	AIC	GoF (p-value)
Clayton	0.2376 (0.0920)	-43.6005	0.9125 (0.0000)
Gumbel	1.2364 (0.0350)	-79.4680	0.2910 (0.0041)
Frank	2.4035 (0.2560)	-85.3951	0.1178 (0.0659)
Gaussian	0.2616 (0.0310)	-58.7777	0.1691 (0.1644)
Student’s *t*	0.3221 (0.0370)	**-107.0723**	0.0985 (**0.4384**)
	5.5976 (1.0060)		

Note: The estimation for parameter of Archimedean and elliptical copulas is carried out through maximum likelihood method whilst the goodness-of-fit test uses Cramér–von Mises statistic. The lowest value of AIC and the highest p-value are in boldface.

**Fig 14:**
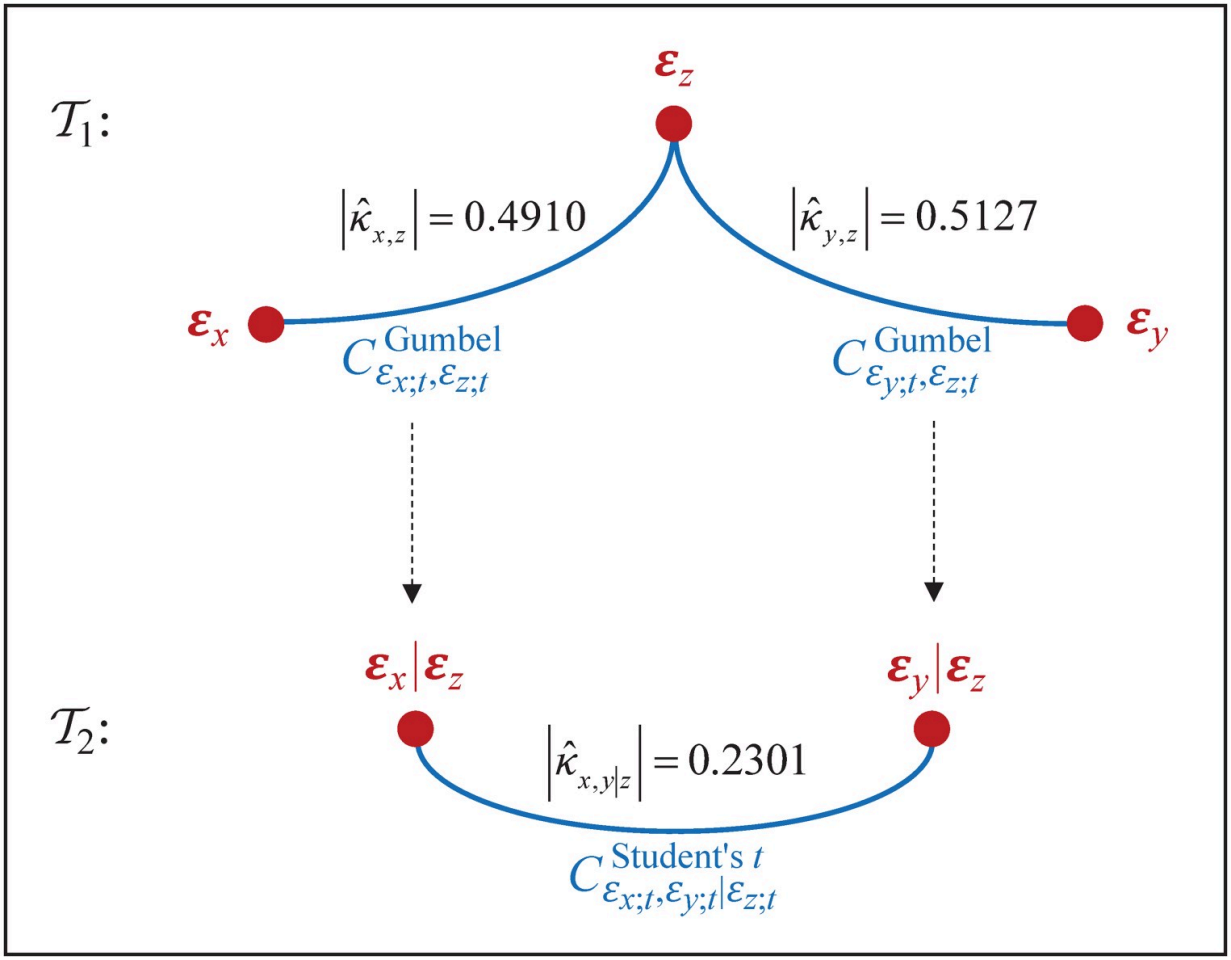
Representation of joint distribution for innovations. The joint distribution for $\left\{\left({\hat{\varepsilon }}_{x;t},{\hat{\varepsilon }}_{y;t},{\hat{\varepsilon }}_{z;t}\right)\right\}$ is represented through (weighted) graph structure of vine copula $\left(F,V_{3},C,K\right)$.

**Fig 15:**
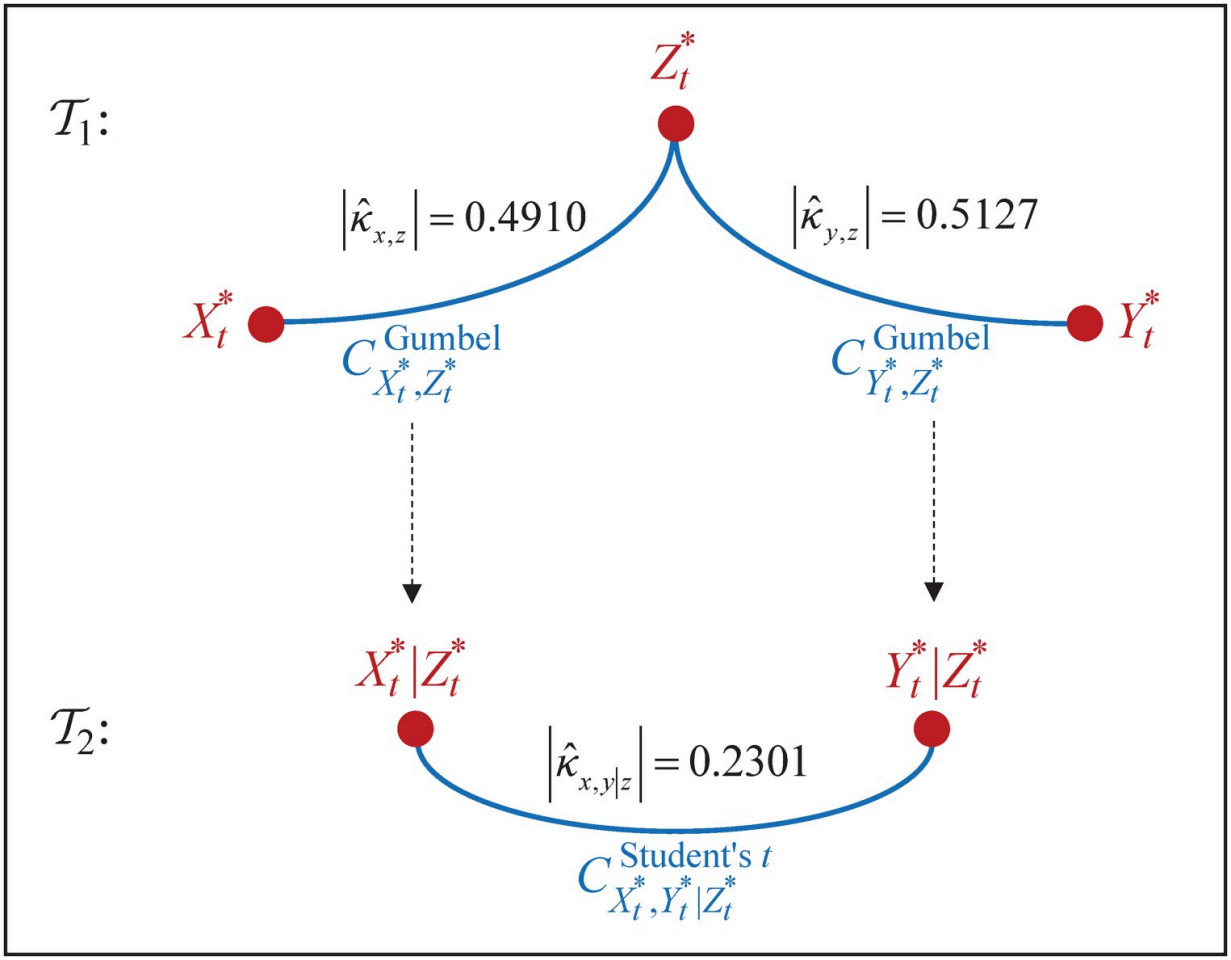
Representation of joint distribution for returns. The joint distribution for $\left(X_{t}^{*},Y_{t}^{*},Z_{t}^{*}\right)=(X_{t}|F_{x;t-1},Y_{t}|F_{y;t-1},Z_{t}\left|F_{z;t-1}\right)$ is represented through (weighted) graph structure of vine copula $\left(F,V_{3},C,K\right)$.

By merging the results above, the best dependence model for (*ε*_*x*;*t*_, *ε*_*y*;*t*_, *ε*_*z*;*t*_) based on the data of innovation estimates $\left\{\left({\hat{\varepsilon }}_{x;t},{\hat{\varepsilon }}_{y;t},{\hat{\varepsilon }}_{z;t}\right)\right\}$ has joint probability function

$$\begin{array}{c} f_{\varepsilon_{x;t},\varepsilon_{y;t},\varepsilon_{z;t}}=f_{\varepsilon_{x;t}}\cdot f_{\varepsilon_{y;t}}\cdot f_{\varepsilon_{z;t}}\cdot c_{\varepsilon_{x;t},\varepsilon_{z;t}}^{\text{Gumbel}}\cdot c_{\varepsilon_{y;t},\varepsilon_{z;t}}^{\text{Gumbel}}\cdot c_{\varepsilon_{x;t},\varepsilon_{y;t}|\varepsilon_{z;t}}^{\text{Student}\prime \text{s}\;t}, \end{array}$$

where $f_{\varepsilon_{x;t}}$, $f_{\varepsilon_{y;t}}$, $f_{\varepsilon_{z;t}}$ are probability function of standard Student’s *t* with degrees of freedom, respectively, ${\hat{\nu }}_{x}$, ${\hat{\nu }}_{y}$, ${\hat{\nu }}_{z}$. Fig 14 shows its graph structure. As a consequence, dependence model $\left(X_{t}^{*},Y_{t}^{*},Z_{t}^{*}\right)$ through graph structure is presented in Fig 15.

### VaR forecast

Now, the one-step-ahead VaR forecasts for Bitcoin, Ethereum and Litecoin returns based on (a)symmetric GARCH(1,1) model are provided in Table 9. Such VaR forecasts are calculated at 95%, 97% and 99% confidence levels (CLs) under two assumptions: (i) perfect dependence, as benchmark, and (ii) vine copula-based dependence among different returns. For the former assumption, we simulate perfectly dependent innovations to calculate the previous returns and volatility and, hence, to find the individual VaR forecasts. This procedure is similarly applied for the latter assumption for which the innovations are simulated through vine copula we have previously determined. Note that the individual VaR forecasts are found simultaneously by Eq (16) for one day only.

**Table 9:** VaR forecasts of marginal risks for one day along with backtesting based on (a)symmetric GARCH(1,1) model under perfect dependence and vine copula-based dependence assumptions.

Model	Currency	CL	VaR	CP	AE	CC (p-value)	QL (Ratio)
Under perfect dependence assumption							
Symmetric	BTC	95%	0.0237	95.24%	0.9517	0.3417 (**0.8429**)	0.00199
		97%	0.0297	97.52%	0.8276	4.7255 (**0.0942**)	0.00149
		99%	0.0456	99.24%	0.7586	4.2226 (**0.1211**)	0.00084
	ETH	95%	0.0462	96.69%	0.6624	12.6247 (0.0018)	0.00365
		97%	0.0617	98.27%	0.5751	12.7249 (0.0017)	0.00280
		99%	0.1103	99.45%	0.5521	8.0716 (0.0177)	0.00159
	LTC	95%	0.0285	96.41%	0.7177	8.7266 (0.0127)	0.00254
		97%	0.0380	98.27%	0.5751	12.7249 (0.0017)	0.00202
		99%	0.0680	99.38%	0.6211	6.5150 (0.0385)	0.00127
Asymmetric	BTC	95%	0.0238	95.24%	0.9517	0.3417 (**0.8429**)	0.00200
		97%	0.0303	97.52%	0.8276	4.7255 (**0.0942**)	0.00149
		99%	0.0466	99.24%	0.7586	4.2226 (**0.1211**)	0.00084
	ETH	95%	0.0459	96.55%	0.6901	10.5727 (0.0051)	0.00362
		97%	0.0613	98.27%	0.5751	12.7249 (0.0017)	0.00278
		99%	0.1096	99.45%	0.5521	8.0716 (0.0177)	0.00157
	LTC	95%	0.0285	96.41%	0.7177	8.7266 (0.0127)	0.00254
		97%	0.0380	98.27%	0.6671	12.7249 (0.0017)	0.00202
		99%	0.0680	99.38%	0.6211	6.5150 (0.0385)	0.00127
Under vine copula-based dependence assumption							
Symmetric	BTC	95%	0.0230	94.98%	1.0041	0.1441 (**0.9305**)	0.00192 (**0.9640**)
		97%	0.0288	96.97%	1.0087	0.0995 (**0.9515**)	0.00141 (**0.9493**)
		99%	0.0443	98.97%	1.0316	0.3272 (**0.8491**)	0.00068 (**0.8107**)
	ETH	95%	0.0458	95.59%	0.8822	1.1211 (**0.5709**)	0.00362 (**0.9910**)
		97%	0.0611	97.79%	0.7351	3.5742 (**0.1674**)	0.00271 (**0.9657**)
		99%	0.1091	99.31%	0.6892	1.7278 (**0.4215**)	0.00135 (**0.8469**)
	LTC	95%	0.0276	96.01%	0.7989	3.3590 (**0.1865**)	0.00219 (**0.8621**)
		97%	0.0369	98.07%	0.6428	6.8704 (0.0322)	0.00165 (**0.8154**)
		99%	0.0659	99.17%	0.8264	3.4289 (**0.1801**)	0.00083 (**0.6492**)
Asymmetric	BTC	95%	0.0233	94.98%	1.0041	0.1441 (**0.9305**)	0.00194 (**0.9686**)
		97%	0.0292	96.97%	1.0087	0.0995 (**0.9515**)	0.00143 (**0.9564**)
		99%	0.0449	98.83%	1.1692	0.8009 (**0.6700**)	0.00069 (**0.8202**)
	ETH	95%	0.0457	94.70%	1.0599	1.7564 (**0.4155**)	0.00360 (**0.9947**)
		97%	0.0610	97.25%	0.9176	2.9389 (**0.2300**)	0.00269 (**0.9698**)
		99%	0.1090	99.24%	0.7571	2.5563 (**0.2786**)	0.00133 (**0.8505**)
	LTC	95%	0.0276	96.01%	0.7989	3.3590 (**0.1865**)	0.00219 (**0.8621**)
		97%	0.0369	98.07%	0.6428	6.8704 (0.0322)	0.00165 (**0.8154**)
		99%	0.0659	99.17%	0.8264	3.4289 (**0.1801**)	0.00083 (**0.6492**)

Note: The one-step-ahead individual VaR forecasts for BTC, ETH and LTC returns are found simultaneously for one day only based on simulated innovations through perfect-dependence copula (for the first assumption) and through vine copula (for the second assumption). The resulted p-value of CC test in boldface is higher than 5% significance level. Meanwhile, the QL ratio in boldface is below one, which means that vine copula-based forecasting procedure performs better.

The forecast accuracy under two assumptions above is assessed and compared through several backtesting methods adopted from Syuhada [34] and Jiménez et al. [20]. These methods include coverage probability (CP), actual over expected (AE) ratio, conditional coverage (CC) test and quantile loss (QL) ratio.

We find that the VaR forecasts for returns by assuming vine copula-based dependence have higher forecast accuracy. This is because the CP for these VaR forecasts are closer to the corresponding CL and their AE ratio are closer to one. Furthermore, this assumption successfully passes the CC test since the resulted p-value is higher than 5% significance level, except for Litecoin at the 97% CL with p-value 0.0322. The choice of significance level may be relaxed to 1% so that the CC test for Litecoin at such CL is also passed. Such results are obtained based on both symmetric and asymmetric GARCH(1,1) models. These show the positive impact of vine copula-based dependence on the VaR forecasts. These are confirmed from the low value of all QL ratios below one. As the CL increases, the QL ratio is getting lower. Meanwhile, the consideration of using asymmetric model do not give better impact on the VaR forecasts. This is because the VaR forecasts derived from both symmetric and asymmetric models do not obviously differ, but the asymmetric model provides higher value of QL ratio.

**Fig 16:**
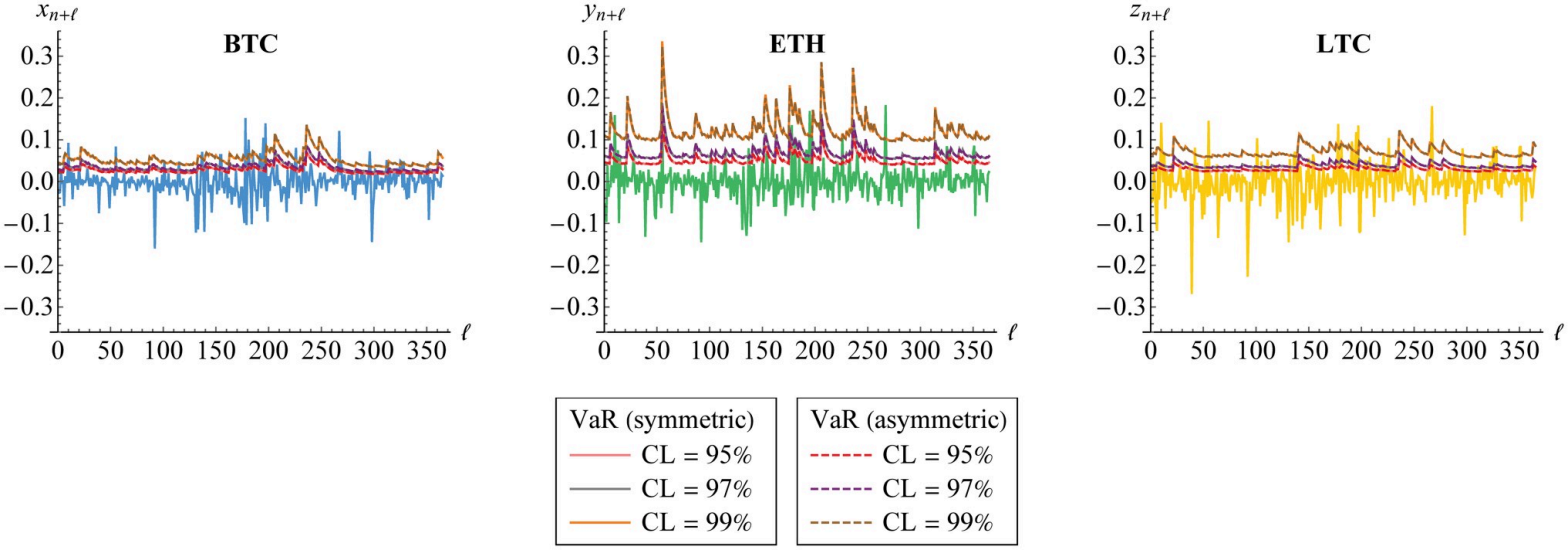
VaR forecasts of marginal risks for several steps/days. The forecasts are derived at 95%, 97% and 99% confidence levels (CLs) based on symmetric (solid line) and asymmetric (dashed line) GARCH(1,1) models for Bitcoin (BTC), Ethereum (ETH) and Litecoin (LTC) through vine copula. They are also compared to the real data.

As mentioned before, we have used the empirical data from 1-1-17 till 31-12-18. Now, we compare the VaR forecasts for several steps/days to the empirical data from 1-1-19 till 31-12-19 as visualized in Fig 16. The comparison is made based on (a)symmetric GARCH(1,1) model where the VaR forecasts are calculated simultaneously by Eq (20) through vine copula.

According to the result in Table 9, we now provide, in Table 10, the one-step-ahead forecast of portfolio risk by using aggregate VaR (aggVaR) through vine copula in comparison to the simple sum of individual VaRs (simplesumVaR), as the benchmark, for one day only. Their forecast accuracy is compared in terms of CP, AE ratio, CC test and QL ratio. We may observe that the aggVaR forecast is lower in value with better accuracy at each CL. The CP as well as AE ratio are closer to the target and the CC test is successfully passed at 5% significance level. Meanwhile, in calculating simplesumVaR, the rejection of null hypothesis of the CC test is only at low level of significance, e.g. 1%, due to the low p-value. Furthermore, the QL ratio between aggVaR and simplesumVaR is below one and getting lower as the CL increases. These show that calculating simplesumVaR is too conservative and overestimates the portfolio risk. In other words, vine copula-based forecasting procedure outperforms perfect dependence assumption. The use of vine copula also makes the portfolio diversified, with diversification coefficient (DC) about 18%. Considering diversification typically provides a stable portfolio since overall risk of the portfolio is properly reduced and, hence, risk allocation is allowed. However, as shown in Table 10, the DC is getting lower as the CL increases. This means that the higher the CL, the less stable the portfolio. The results under symmetric and asymmetric GARCH(1,1) models are in line. However, the asymmetric one performs worse with higher value of the QL ratio although the resulted DC is higher.

**Table 10:** VaR forecasts of portfolio risk for one day along with backtesting and diversification coefficient based on (a)symmetric GARCH(1,1) model.

Model	CL	VaR Forecast		DC%	CP	AE	CC (p-value)	QL (Ratio)
Symmetric	95%	simplesum	0.0964	17.95	96.27%	0.7453	7.0780 (0.0290)	0.00812
		aggVaR	0.0791		94.73%	1.0548	1.1926 (**0.5508**)	0.00719 (**0.8856**)
	97%	simplesum	0.1267	17.80	97.93%	0.6901	6.8669 (0.0323)	0.00627
		aggVaR	0.1042		97.15%	0.9484	0.1473 (**0.9290**)	0.00534 (**0.8517**)
	99%	simplesum	0.2194	17.42	99.38%	0.6211	6.5150 (0.0385)	0.00368
		aggVaR	0.1812		98.89%	1.1103	2.0685 (**0.3555**)	0.00265 (**0.7193**)
Asymmetric	95%	simplesum	0.0966	17.99	96.27%	0.7453	7.0780 (0.0290)	0.00811
		aggVaR	0.0792		94.73%	1.0548	1.1926 (**0.5508**)	0.00719 (**0.8872**)
	97%	simplesum	0.1271	17.84	97.93%	0.6901	6.8669 (0.0323)	0.00625
		aggVaR	0.1044		97.15%	0.9484	0.1473 (**0.9290**)	0.00533 (**0.8534**)
	99%	simplesum	0.2198	17.46	99.38%	0.6211	6.5150 (0.0385)	0.00366
		aggVaR	0.1815		98.89%	1.1103	2.0685 (**0.3555**)	0.00264 (**0.7207**)

Note: The aggregate VaR forecast is calculated for one day only through vine copula and is compared to the simple sum of individual VaR forecasts. The diversification coefficient is also calculated. The resulted p-value of CC test in boldface is higher than 5% significance level whilst the QL ratio in boldface is below one.

**Fig 17:**
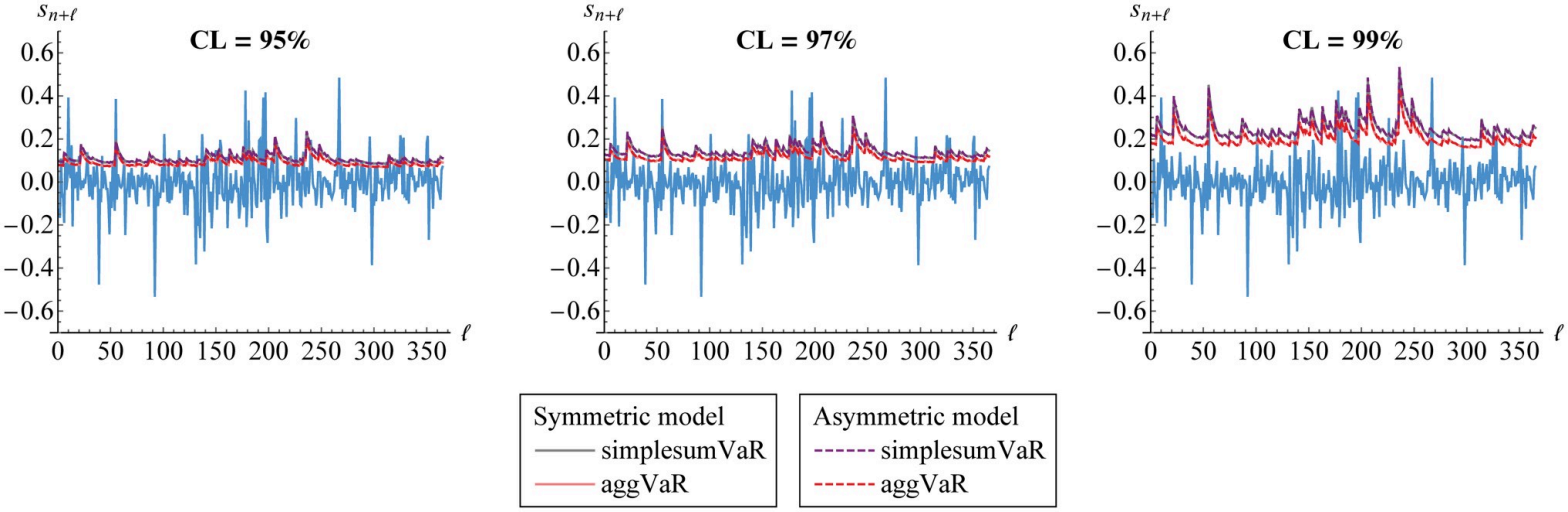
VaR forecasts of portfolio risk for several steps/days. The aggVaR forecasts are calculated at 95%, 97% and 99% confidence levels (CLs) based on symmetric (solid line) and asymmetric (dashed line) models through vine copula. They are compared to the simplesumVaR (purple) and to the real data (blue).

**Fig 18:**
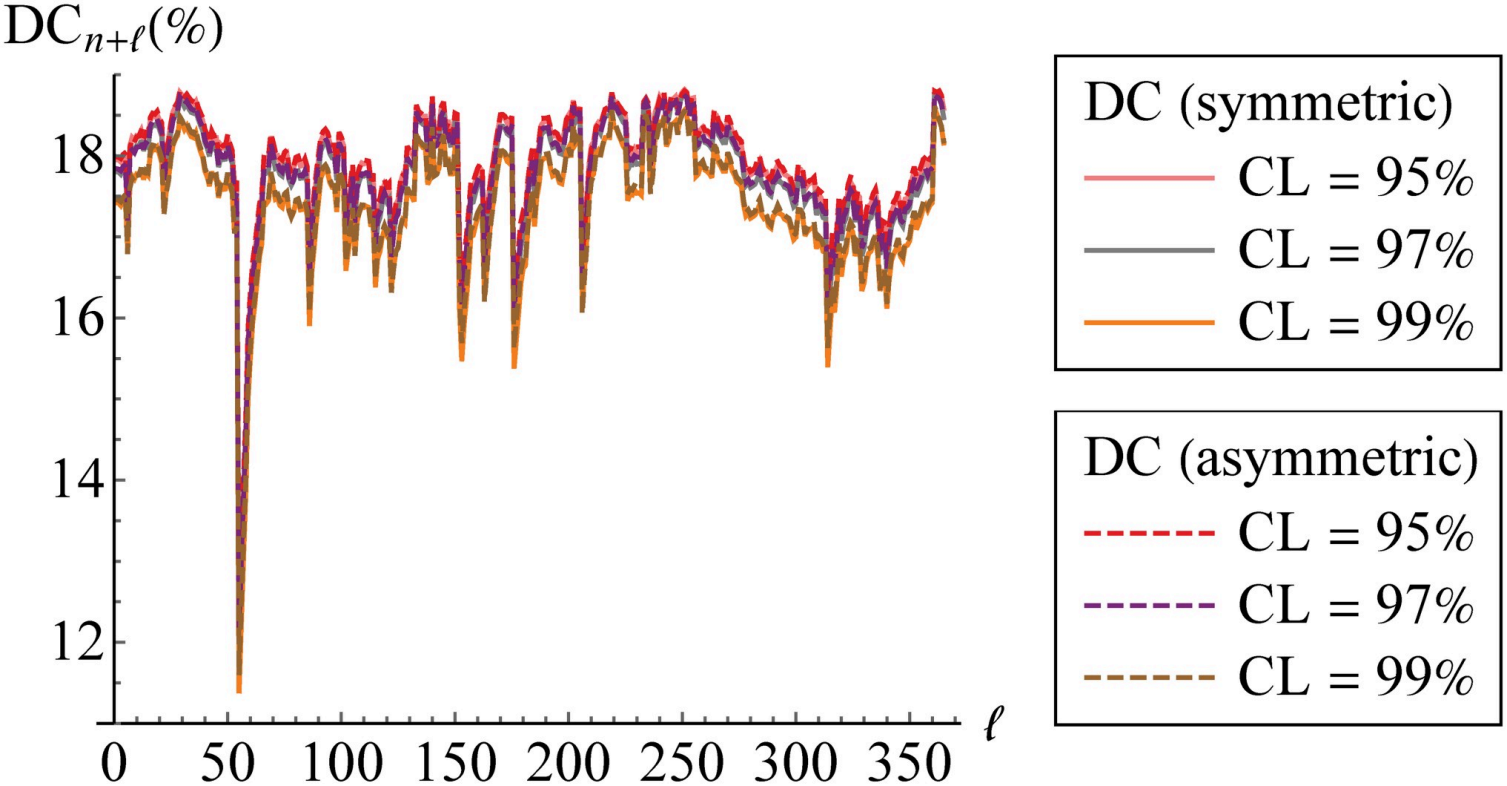
Diversification coefficients of aggregate VaR forecasts. The diversification coefficients (DCs) are calculated at 95%, 97% and 99% confidence levels (CLs) based on symmetric (solid line) and asymmetric (dashed line) models through vine copula.

In addition, we calculate the aggVaR forecasts for several steps/days. Such forecasts are visualized in Fig 17. This figure also provides their comparison to the simplesumVaR and the real data of aggregated returns from 1-1-19 till 31-12-19. Meanwhile, the corresponding DCs are displayed in Fig 18.

## Conclusion

Model for dependent risks that form a portfolio risk has been constructed. Through vine copula, their marginal risks assumed to follow GARCH(1,1) model are coupled with complete representation through graph structure. This approach provides high flexible pair-copula model since it is able to capture dependence structure of all possible pairs of risks by using different bivariate copulas with the best criterion. As we hope, applying this dependence model to forecast VaR for Bitcoin, Ethereum and Litecoin returns provides good forecast accuracy. Furthermore, the resulted portfolio VaR forecast is low in value with high accuracy instead of simply summing the individual VaR forecasts under perfect dependence assumption. As a consequence, the portfolio is well diversified which means that its overall risk is well managed and reduced. The results under consideration of both symmetrical volatility and asymmetrical volatility in the marginal model are in line. However, the asymmetric model does not perform better although it makes the portfolio more diversified.

For further research, modeling dependence may be carried out for higher-dimensional risk data due to the increasing number of risks in the cryptocurrency market or other markets nowadays. Furthermore, the marginal risk model may be extended to another observable volatility models of GARCH. The use of latent volatility model of Stochastic Volatility Autoregressive (SVAR), see e.g. Han et al. [54] and Syuhada [34], perhaps gives more interesting results. This is due to volatility shock that appears in volatility process. As for risk measure forecast, it is important to consider (i) expected-based risk measure, namely Expected Shortfall or Tail-VaR, and (ii) expectile-based risk measure of EVaR [51]. Whilst the former (VaR) is more probability-based risk measure, the latter (ES/TVaR, EVaR) takes the magnitude of losses into account.

## Supporting information

S1 Data (XLSX)

S1 File (PDF)
