# Supplementary material for: Modeling risk dependence and portfolio VaR forecast through vine copula for cryptocurrencies
Source: PLoS One. 2020 Dec 23;15(12):e0242102. doi: 10.1371/journal.pone.0242102 (PMC7757910; doi:10.1371/journal.pone.0242102)
Supplement: S1 File — (PDF) [file pone.0242102.s002.pdf]

Modeling risk dependence and portfolio VaR forecast through vine copula for cryptocurrencies

Khreshna Syuhada[1*], Arief Hakim[1]

1 Statistics Research Division, Institut Teknologi Bandung, Indonesia

* khreshna@math.itb.ac.id

Data Availability Statement

All relevant data are within the manuscript and the supporting information file S1 Data. Data are from Coin Market Cap (coinmarketcap.com) for period 1 January 2017 till 31 December 2018 (730 days).
